# Deficiency of Ku Induces Host Cell Exploitation in Human Cancer Cells

**DOI:** 10.3389/fcell.2021.651818

**Published:** 2021-03-29

**Authors:** Okay Saydam, Nurten Saydam

**Affiliations:** ^1^Department of Pediatrics, University of Minnesota, Minneapolis, MN, United States; ^2^Department of Biochemistry, Molecular Biology and Biophysics, University of Minnesota, Minneapolis, MN, United States

**Keywords:** Ku protein, Ku70/Ku86, cancer metastasis, cell invasion, genome instability, ERM proteins, extracellular vesicles

## Abstract

Cancer metastasis is the major cause of death from cancer (Massague and Obenauf, 2016; Steeg, 2016). The extensive genetic heterogeneity and cellular plasticity of metastatic tumors set a prime barrier for the current cancer treatment protocols (Boumahdi and de Sauvage, 2020). In addition, acquired therapy resistance has become an insurmountable obstacle that abolishes the beneficial effects of numerous anti-cancer regimens (De Angelis et al., 2019; Boumahdi and de Sauvage, 2020). Here we report that deficiency of Ku leads to the exploitation of host cells in human cancer cell line models. We found that, upon conditional deletion of *XRCC6* that codes for Ku70, HCT116 human colorectal cancer cells gain a parasitic lifestyle that is characterized by the continuous cycle of host cell exploitation. We also found that DAOY cells, a human medulloblastoma cell line, innately lack nuclear Ku70/Ku86 proteins and utilize the host-cell invasion/exit mechanism for maintenance of their survival, similarly to the Ku70 conditionally-null HCT116 cells. Our study demonstrates that a functional loss of Ku protein promotes an adaptive, opportunistic switch to a parasitic lifestyle in human cancer cells, providing evidence for a previously unknown mechanism of cell survival in response to severe genomic stress. We anticipate that our study will bring a new perspective for understanding the mechanisms of cancer cell evolution, leading to a shift in the current concepts of cancer therapy protocols directed to the prevention of cancer metastasis and therapy resistance.

## Significance

Cancer cell plasticity and therapy resistance have been major obstacles in modern cancer medicine. Development of effectual cancer therapeutics requires understanding of well-defined mechanisms of cancer cell evolution. Here, we provide definitive evidence for an unconventional mechanism of cancer cell adaptation by which the cells critically deficient for Ku protein acquire a parasitic lifestyle and exploit the neighboring host cells. Using a genetically engineered human colorectal cancer cell line and a naturally occurring medulloblastoma tumor cells, we showed that the lack of Ku70/Ku86 leads to parasitic cell invasion and exploitation of neighboring cells. These findings point toward a previously unknown mechanism of cancer cell adaptation, and support the critical significance of tumor cell-host cell interactions in the course of cancer dissemination and therapy resistance.

## Introduction

Tumor cell plasticity is a key challenge for current cancer therapy (Boumahdi and de Sauvage, 2020). Extensive genetic heterogeneity within tumors reinforces tumor evolution, and thereby supports metastatic spread and drug resistance of cancer cells (McGranahan and Swanton, 2017; Dagogo-Jack and Shaw, 2018; Kyrochristos et al., 2019). Understanding the processes yielding profound genetic diversity within the same tumor, as well as between the primary and metastatic pairs is a fundamental issue and of immediate research interest while cancer cell metastasis and drug resistance are winning out and offsetting the current cancer therapy protocols at large.

Ku is a highly abundant DNA binding protein that exists as a stable heterodimer consisting of two subunits, Ku70 and Ku86. Ku70/Ku86 heterodimer is best characterized for its ability to recognize and bind the broken ends of double-strand DNA (Walker et al., 2001). The functional importance of Ku resides in its key role in the non-homologous end joining (NHEJ) repair of DNA double-strand breaks (DSBs), and also in the protection of telomeres (Fell and Schild-Poulter, 2015). While Ku is not essential for organismal development in mice (Nussenzweig et al., 1996; Zhu et al., 1996; Gu et al., 1997; Nussenzweig et al., 1997; Manis et al., 1998), its function has been reported to be critical for the maintenance of genome integrity and cell survival in humans (Li et al., 2002; Fattah et al., 2008).

In this study, we discovered that loss of functional Ku protein leads to a striking and uncommon shift in the cell-to-cell interaction dynamics of two different human cancer cell lines, a colorectal cancer cell line HCT116 and a desmoplastic cerebellar medulloblastoma cell line DAOY, which is associated with the formation of an aggressive cell type that is able to invade the neighboring cells, followed by the departure from the host as an intact single cell. To our knowledge, this is the first report describing a previously unknown mechanism of an acquired parasitic life style of cancer cells in response to the functional loss of an essential gene in human cells.

## Materials and Methods

### Cell Lines

HCT116 (ATCC^®^ CCL-247^TM^) cells, a colorectal cancer cell line, were purchased from the ATCC. Ku70 conditionally-null HCT116 cell line was a gift from Eric A. Hendrickson (Fattah et al., 2008). Briefly, these cells were generated using a Cre/LoxP recombination approach (Sauer and Henderson, 1988). First, exon 4 of Ku70 was replaced by its “floxed” equivalent using a rAAV-mediated targeting strategy to generate a Ku70^f/+^ HCT116 cell line. The second allele of Ku70 was targeted using a similar rAAV-mediated method except that the donor DNA contained a floxed NEO-drug resistance cassette resulting in a Ku70^f/Neo^ HCT116 cell line. CreER was then introduced into these cells using the plasmid system of CreER-t2-pcDNA3.1-Puromycin, and treated with 10 nM 4-hydroxytamoxifen (4-OHT) to acquire Ku70^f/–^ HCT116 cells, which were no longer G418-resistant. These Ku70^f/–^ cells were then subjected to a second round of 4-OHT treatment (10 nM) to generate Ku70^null^ cells (Fattah et al., 2008). DAOY cells, derived from a desmoplastic cerebellar medulloblastoma, were purchased from the ATCC (ATCC^®^ HTB-186^TM^). HEK293T cells (ATCC^®^ CRL-3216^TM^) were purchased from the ATCC and used for viral packaging.

### Lentiviral Plasmid Construction, Viral Particle Production, and Infections

pHIV-H2B-eGFP plasmid was a gift from Maria Pia Cosma (Addgene plasmid # 91776) (Sottile et al., 2016). pHIV-H2B-mRFP plasmid was a gift from Bryan Welm and Zena Werb (Addgene plasmid # 18982) (Welm et al., 2008). Palm-EGFP expressing lentiviral vector (CS-B0093-LV105-01) and Palm-tdTomato (CS-B0093-LV105-01) expressing lentiviral vectors were constructed as follows: A gene block consisting of a 60 bp ORF of human GAP43 gene (NM_002045.3) [codes for a palmitoylation signal (Palm)] fused to the N-terminus of an EGFP reporter gene (Palm-EGFP) or to the N-terminus of a tdTomato reporter gene (Palm-tdTomato) was cloned into the pReceiver-Lv105 lentiviral vector (Genecopoeia). For RNAi dowregulation of Ku70 (XRCC6) in DAOY cells, we used the SMARTvector inducible shRNA expression system (Dharmacon, SMARTvector Inducible Human XRCC6 mCMV-TurboRFP shRNA, V3SH11252-226496333). Following lentiviral induction and drug selection, DAOY cells were treated with 1 μg/ml doxycycline hyclate (Thermo Scientific, Cat #ICN19895510) for 5 days in order to induce the expression of Ku70 shRNAs.

Lentiviruses were produced by cotransfection of HEK293T cells with the lentiviral constructs and packaging plasmids psPAX2 (Addgene #12260) and pMD2.G (Addgene #12259). Transfections were carried out with Lipofectamin 2000 (Invitrogen). Viral particles were harvested at 48 and 72 h posttransfection. HCT116 cells and DAOY cells were infected with the lentiviruses in the presence of 10 μg/mL of polybrene (Sigma) for 24 h. Three days after the transduction, DAOY cells were selected with 1 μg/mL puromycin (GIBCO) for 5 days.

### Western Blot

HCT116 wild type cells and HCT116 Ku70^f/–^ cells (clone no. #6.2) were left untreated or treated with 10 nM 4-OHT for 10 days. Cells were harvested and subjected to Western blot as described earlier (Strobel et al., 2017). Ku70 was detected with a rabbit polyclonal Ku70 antibody (sc-9033), and Ku86 was detected with a mouse monoclonal Ku86 antibody (sc-5280). β-actin served as loading control.

### Live-Cell Imaging

HCT116 Ku70^f/–^ cells were infected with lentiviruses expressing H2B-EGFP or H2B-mRFP, and propagated for 1 week. These cells were further infected with the lentiviruses expressing either Palm-EGFP or Palm-tdTomato. One week later, the Ku70^f/–^ cells infected with these lentiviruses were treated with 10 nM 4-OHT (Sigma H7904) for 5 days. One day before the imaging, the Ku70^null^ cells expressing H2B-EGFP and Palm-EGFP were co-cultured (1:1 ratio) with the Ku70^null^ cells expressing H2B-RFP and Palm-tdTomato, and subjected to live-cell imaging for 48 h. The live-cell imaging studies were performed by using a Nikon BioStation IM microscope system that enables long term time-lapse experiments for phase contrast and fluorescence imaging (green and red excitation) of live cells. Of note, after the lentiviral infections, no drug selection was performed because of the lack of drug resistance gene in the pHIV-H2B-EGFP and pHIV-H2B-mRFP vectors. In addition, HCT116 Ku70^f/–^ cells are not compatible for the selection of Palm-EGFP and Palm-tdTomato expressing cells with puromycin, because of the stable expression of the CreER-t2-pcDNA3.1-Puromycin system in these cells. Therefore, only a small fraction of the cells expressing Palm-EGFP or Palm-RFP was visible under our live-cell imaging settings, while the strong fluorescent signals of the H2B-EGFP or H2B-mRFP reporters were dominant in the majority of the cells. The time lapse imaging was performed using the time intervals of 4 min (for DAOY cells) and 5 min (for HCT116 Ku70^null^ cells) for duration of 48 h. At least 20 different areas were set to be imaged per sample. At least 20 time-lapse recordings per sample were analyzed, and the movies were generated in the AVI format by using FIJI software. Similarly, DAOY cells were infected with the Palm-EGFP or Palm-tdTomato expressing lentiviruses, and selected with puromycin (1 μg/ml) for 5 days prior to the live-cell imaging for 48 h.

### Immunofluorescence

HCT116 WT and Ku70^f/–^ cells were either left untreated or treated with 10 nM 4-OHT for 10 days, fixed with 4% formaldehyde and subjected to immunofluorescence analysis as described earlier (Strobel et al., 2017). Cells were immuno-stained with a rabbit monoclonal antibody for phospho-ERM proteins (Cell Signaling, #3726). DNA was stained with DAPI (Vectashield, H-1200), and cells were visualized by the Olympus FluoView FV1000 BX2 Upright Confocal System.

For the confocal microscope examination of the live-cell imaging samples, following the time-lapse imaging, cells were fixed with 4% formaldehyde subsequently, and DNA was labeled with DAPI. Cells were then visualized with the Olympus FluoView FV1000 IX2 inverted confocal microscopy system.

DAOY cells were fixed with 4% formaldehyde and immuno-stained with a mouse monoclonal antibody for Ku70 (Santa Cruz, sc-17789) and/or a rabbit monoclonal antibody for phospho-ERMs (Cell Signaling, #3726). DNA was labeled with DAPI, and cells were visualized with the Olympus FluoView FV1000 BX2 Upright Confocal System.

DAOY cells were infected with the Palm-EGFP or Palm-tdTomato expressing lentiviruses. After the drug selection with puromycin (1 μg/ml) for 5 days, cells were fixed with 4% formaldehyde. The cells expressing Palm-tdTomato were directly visualized after the staining of nuclei with DAPI. DAOY cells expressing Palm-EGFP were subjected to immuno-staining with an antibody for phospho-ERM (Cell Signaling, #3726), and visualized using an Olympus FluoView FV1000 BX2 Upright Confocal System. All images taken from the confocal microscopy examinations were analyzed by using the Adobe Photoshop CS5 software.

## Results

**FIGURE 1:**
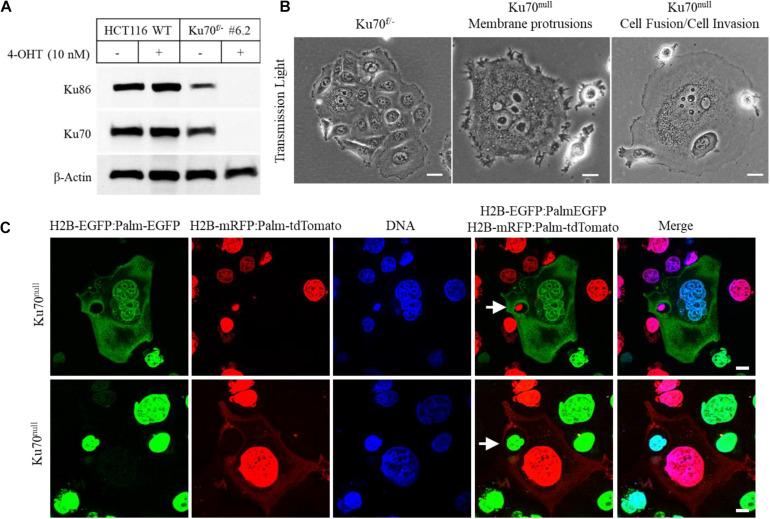
Conditional loss of Ku70 in HCT116 cells leads to cell invasion. Ku70 and Ku86 were detected in the cell extracts of wild-type (WT) and engineered HCT116 cells (Ku70^f/–^ cells; clone no. #6.2) following the treatment with 10 nM 4-OHT for 10 days to obtain conditionally-null Ku70 (Ku70^null^) cells **(A)**. Phase contrast images of HCT116 Ku70^f/–^ and Ku70^null^ cells showing formation of giant cells, elongated membrane protrusions, cell fusions, cell invasion, and cell-in-cell structures at Day10 of 4-OHT treatment. Scale bar, 20 μm **(B)**. Confocal microscopy examination of Ku70^null^ cells following live-cell imaging. HCT116 Ku70^f/–^ cells expressing H2B-EGFP:Palm-EGFP or H2B-mRFP:Palm-tdTomato were treated with 10 nM 4-OHT for 5 days and co-cultured at 1:1 ratio 1 day prior to the imaging. Following the live-cell imaging for 48 h, cells were fixed and examined via a confocal microscope. Two different examples of giant host cells with engulfed nuclei/cell (white arrows) are shown. Scale bar, 10 μm **(C)**.

In this study, we investigated the cellular dynamics of a human colorectal cancer cell line (HCT116) that is engineered for conditional deletion of *XRCC6* gene (codes for Ku70 protein) (Fattah et al., 2008) by using live-cell imaging and confocal microscopy technologies. The conditional loss of Ku70 and its obligate partner Ku86 was achieved following the treatment of Ku70-heterozygous cells (Ku70^f/–^) with 10 nM 4-hydroxytamoxifen (4-OHT) (Figure 1A). Light microscopic investigation of Ku70^f/–^ cells treated with 4-OHT for 10 days showed that the surviving fraction of these cells (∼10–20% of initially seeded cells) exhibited frequent formation of giant cells, widespread emergence of elongated membrane protrusions, cell-in-cell structures, and a phenotype of “attacking cells” that are juxta-positioned or attached to “host cells” (Noubissi and Ogle, 2016; Figure 1B). To be able to track actively moving cells, the nuclei of Ku70^f/–^ cells were labeled by using the lentiviral H2B-EGFP or H2B-mRFP reporters, and the cell membranes of Ku70^f/–^ cells were labeled by the membrane-targeted EGFP (Palm-EGFP) or tdTomato (Palm-tdTomato) reporters that are fused to a palmitoylation signal (Palm) (Liu et al., 1994; Lai et al., 2015). The Ku70^f/–^ cells expressing H2B-EGFP:Palm-EGFP or H2B-mRFP:Palm-tdTomato were subjected to live-cell imaging after the treatment with 10 nM 4-OHT for 5 days that was followed up with the co-culturing of the green and red fluorescent cells at 1:1 ratio for 24 h. The live-cell imaging of these cells for duration of 48 h showed that the cells conditionally lacking Ku70 protein (Ku70^null^ cells) are able to exploit the neighboring cells, which is characterized by a cycle of the invasion of the host cell and the subsequent departure from the host cell (Supplementary Movie 1 and Supplementary Figures S1A–D). We also observed that the invading Ku70^null^ cells are able to exit from the host cell via a budding-like membrane detachment mechanism (Supplementary Movie 1 and Supplementary Figures S1A–D). Of note, to avoid the confusion about the terminology of “invasion” that is commonly used to state the aggressive cancer cell behavior, mostly in the context of cancer metastasis, we preferred to use the term of “exploitation” which is used to state the host cell invasion and the survival within the host by utilizing host cell resources in the field of parasitology (Poulin, 2011; Poulin and Randhawa, 2015). Further analyses of these cells right after the live-cell imaging session via a confocal microscope confirmed the formation of cell-in-cell structures as a result of host cell exploitation, as exemplified in Figure 1C (upper panel) demonstrating that an engulfed nucleus marked with H2B-mRFP was detected inside of a giant cell expressing H2B-EGFP and Palm-EGFP. Another example is shown in Figure 1C (lower panel) for a cell nucleus marked with H2B-EGFP encircled by the cytoplasm of a giant cell expressing both H2B-mRFP and palm-tdTomato. Furthermore, we observed that Ku70^null^ cells dynamically move within the host cells, and they are either released from the host as a result of host cell death (Supplementary Movie 2 and Supplementary Figures S2A,B) or they are able to exit from the host via membrane breaching (Supplementary Movie 1 and Supplementary Figures S1A–D). These observations suggest that the HCT116 cells deficient for Ku70/Ku86 heterodimer are able to adopt a parasitic lifestyle and exploit neighboring cells.

In addition, the live-cell imaging of Ku70^null^ HCT116 cells showed that frequent numbers of rounded cells that resemble to mitotic cells were translocated onto or inside the host cell, where they were noticeably motile while interacting with the host cell nuclei through their cell membrane protrusions (Supplementary Movie 3). These observations suggest that Ku70^null^ cells use a host cell-dependent mechanism to facilitate their proliferation.

In line with this idea, we visualized that some attacking cells undergo faulty cell division within the host cell, followed by a subsequent departure from the host (Supplementary Movie 4 and Supplementary Figures S3A,B). Surprisingly, we also observed that Ku70^null^ cells were able to adopt cytoplasm from neighboring cell bodies (Supplementary Movie 4).

Another remarkable phenomenon observed in Ku70^null^ cells was the formation of massive membrane protrusions that are actively spreading out toward the neighboring cells (Supplementary Movie 5). More surprisingly, we detected that some intracellular bodies in the shape of round or oval structures resembling micronuclei were being assembled at the tips of these membrane protrusions (Supplementary Movie 5). Those newly formed bodies were initially contained by a vesicular membrane at the tips of the protrusions, and they were transported in single file through the long, narrow tubes of the plasma membrane toward the cell nucleus as shown in Supplementary Movie 5. The exact characterization of these new assemblies extracted from extracellular milieu requires further investigation.

**FIGURE 2:**
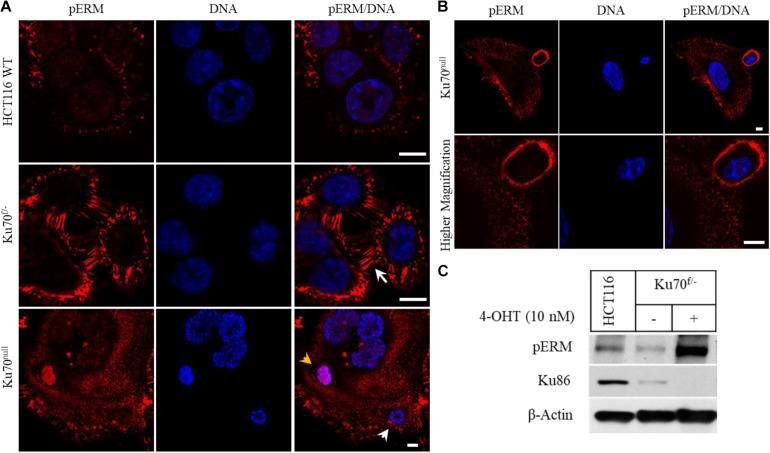
Conditional loss of Ku70 in HCT116 cells leads to accumulation of phospho-ERM at cell membranes. HCT116 WT and Ku70^f/–^ cells were either left untreated or treated with 10 nM 4-OHT for 10 days. Cells were immuno-stained with a phospho-ERM antibody, and DNA labeled with DAPI. White arrow indicates elongated membrane protrusions, and arrow heads indicate engulfed nuclei/cell. The intact plasma membrane of an engulfed cell stained with the phospho-ERM (pERM) antibody is also shown (yellow arrow head). Scale bar, 10 μm **(A)**. A typical “attacking cell” and “host cell” are shown in lower and higher magnification. Scale bar, 10 μm **(B)**. HCT116 WT and Ku70^f/–^ cells were treated as described in **(A)**, and cell extracts were subjected to Western blot for detection of pERM proteins and Ku86. β-Actin served as loading control **(C)**.

To elucidate the mechanisms of parasitic host cell invasion associated with Ku deficiency, we immuno-stained HCT116 wild-type, Ku70^f/–^ and Ku70^null^ cells for ERM proteins (Ezrin, Radixin, Moesin), a group of three related proteins with critical roles in regulation of membrane protrusions, cell migration, and adhesion (Clucas and Valderrama, 2014; Ponuwei, 2016; Kawaguchi et al., 2017). We found that the plasma membranes of Ku70^f/–^ cells that contain only one copy of *XRCC6* gene and express only 50% of normal cellular Ku70 levels were strongly positive for the phosphorylated (active) forms of ERMs (pERM), in comparison to the parental HCT116 cells (Figure 2A). In addition, these cells were detected with elongated microvilli structures at their membranes (white arrow) (around 60% of all cells), while parental cells showed only a moderate pERM staining with no remarkable membrane protrusions (Figure 2A). These observations suggest that 50% decrease in cellular Ku70 is sufficient for the activation of ERMs, which leads to remodeling of the plasma membranes. Yet, we cannot exclude that a leaky CreER expression in this conditional cell line system may be one of the reasons for this phenotype of Ku70^f/–^ cells. Nevertheless, the signal for pERM became much more evident following the treatment of Ku70^f/–^ cells with 10 nM 4-OHT for 6 days, suggesting that conditional loss of Ku70 leads to increased accumulation of active ERMs in ∼90% of surviving cell population. The pERM immunostaining of Ku70^null^ cells also allowed the visualization of the cell-in-cell structures (arrow heads), as shown in the lowest panel of Figure 2A where the intact plasma membrane of an internalized cell is stained with pERM (yellow arrow head). In line with our live-cell imaging, the “attacking cells” were detected with a thick coat of pERM proteins by which they are likely to penetrate into the host (Figure 2B). As expected, the phosphorylation of ERMs at conserved, regulatory threonine positions were highly induced in the cell extracts of Ku70^null^ cells (Figure 2C).

**FIGURE 3:**
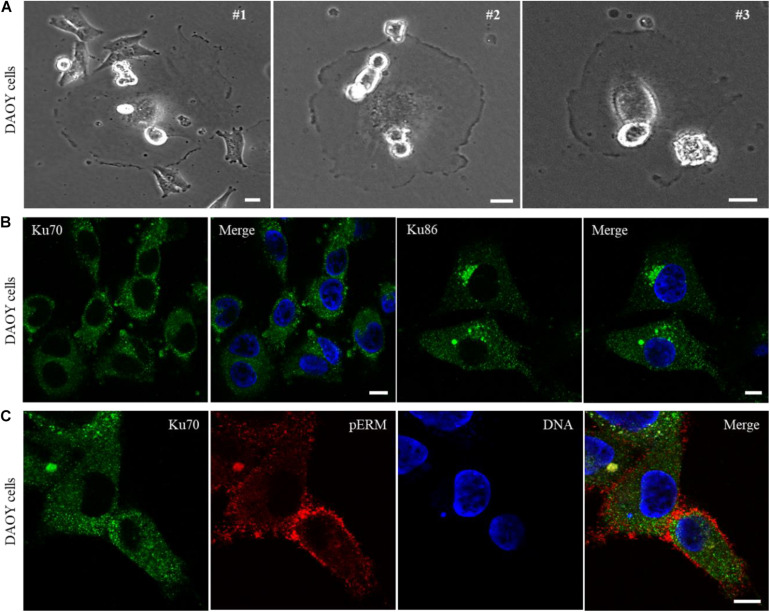
DAOY cells derived from a desmoplastic cerebellar medulloblastoma show explicit exclusion of Ku70/Ku86 from cell nuclei. Light microscopy examination of DAOY cells shows wide-spread appearance of giant cells, with similar phenotypic features of Ku70-lacking HCT116 cells. DAOY cells showing a giant cell surrounded with the smaller size of cells (#1), the rounded/mitotic cells placed on the body of giant cells (#1, #2, #3), and the rounded/mitotic cells fused/penetrated into the giant cell membranes (#1, #2, #3) are shown in **(A)**. Scale bar, 20 μm. Immunostaining of DAOY cells for Ku70 or Ku86 shows nuclear exclusion of Ku70/Ku86. Scale bar, 10 μm **(B)**. DAOY cells were immuno-stained for Ku70 and pERM, and an example of “attacking cell” with a surrounding condensed pERM signal is shown **(C)**. Scale bar, 10 μm.

In a search for the cancer cell lines showing phenotypic similarities to Ku70^null^ cells based on a light microscopy examination, we identified DAOY cell line, derived from a human desmoplastic cerebellar medulloblastoma tumor tissue, showing remarkable similarity to the Ku70 conditionally-null HCT116 cells. DAOY cells were characterized with a large number of giant cells which were mostly surrounded by smaller cells (Figure 3A, #1), lots of rounded/mitotic cells that were predominantly sitting on the body of the giant or non-mitotic cells (Figure 3A, #2), and “attacking cells” that penetrated into the host cell membranes (Figure 3A, #1, #2, #3). Next, we investigated the cellular localization of Ku70 and Ku86 in DAOY cells via immunofluorescence analysis, and found that both Ku70 and Ku86 are excluded from the nuclei of DAOY cells, while both are detectable in the cytoplasm (Figure 3B).

To investigate whether the lack of nuclear Ku leads to the parasitic-type of host cell invasions and host-dependent cell proliferation, the DAOY cells expressing membrane-targeted Palm-EGFP reporter were subjected to live-cell imaging for duration of 48 h. The examination of the time-lapse images demonstrated that DAOY cells are able to exploit neighboring cells, similarly to the Ku70-conditionally null HCT116 cells (Supplementary Movie 6 and Supplementary Figures S4A–H). In addition, we observed that some DAOY cells station onto the host cell body and strives for cell division in a host-cell dependent manner in a frequent base (Supplementary Movie 7 and Supplementary Figures S5A–E). Notably, similar to HCT116 Ku70^null^ cells, DAOY cells showed faulty cytokinesis where daughter cells fail to segregate, and thus form multinucleated cells (Supplementary Movie 8). Of note, given the capacity of the time-lapse imaging system used in this study, making a clear distinction between the cells docking on the host cell membrane and the cells fully immersed into the host cell cytoplasm was not always attainable. However, our observations support both circumstances, which is exemplified in Supplementary Movie 7 showing that a mitotic-like cell that was actively rotating on the body of a giant cell could remain associated with the host cell for at least 26 h (Supplementary Figures S5A–D), prior to a rapid translocation into a neighboring cell. These findings suggest that DAOY cells are likely to operate the intercellular material exchange either by directly attaching to the neighboring cell through a membrane docking system or via an actual invasion of the host. By using an inducible shRNA system targeting Ku70 in DAOY cells, we observed similar phenomenon of host cell exploitation as in the HCT116 Ku70^null^ cells as well as in the intact DAOY cells (Supplementary Movie 9), suggesting that the nuclear exclusion of Ku70/Ku86 is sufficient to induce parasitic cell invasions in DAOY cells. It also suggests that mislocalization of Ku70/Ku86 heterodimer might naturally occur in some cancer cells such as medulloblastoma-derived DAOY cells. In addition, the “attacking cells” penetrated into the host with their rich coat of pERMs were also evident in DAOY cells (Figure 3C), providing further evidence that the lack of nuclear Ku may induce host cell exploitation in DAOY cells, similarly to the HCT116 Ku70^null^ cells.

**FIGURE 4:**
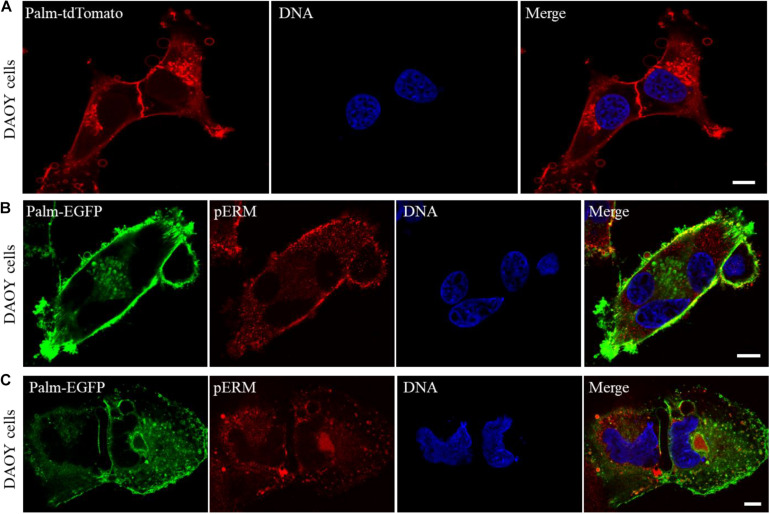
DAOY cells release a large number of vesicles that are loaded with pERM proteins, which may facilitate cell fusion and invasions. Confocal microscope examination of DAOY cells expressing a membrane-targeted Palm-tdTomato reporter shows extensive number of vesicles that surround the cells in varying sizes. Scale bar, 10 μm **(A)**. Interaction of plasma membranes during cell fusion was visualized in Palm-EGFP expressing DAOY cells after immuno-staining with a pERM antibody. Scale bar, 10 μm **(B,C)**. Note that the majority of both intracellular and extracellular vesicles of DAOY cells are loaded with pERM proteins **(B,C)**.

Next, to gain insight into the mechanism of host cell invasions, the membranes of DAOY cells were marked with a membrane-targeted tdTomato reporter (Palm-tdTomato), and examined using a confocal microscope. Our observations showed that DAOY cells release excessive amounts of vesicular bodies that greatly vary in size and can reach up to 5 μm or larger in some cases (Figure 4A). In addition, the examination of the cells expressing palm-EGFP revealed that both extracellular and intracellular vesicular bodies of DAOY cells are packed with pERMs (Figures 4B,C). These observations indicate that pERMs are actively transported between the cells through the extracellular vesicles, yet the exact role of those vesicular bodies in cell fusion and host cell invasion requires further investigation.

## Conclusion

The functional importance of Ku70/Ku86 heterodimer resides in its key role in the NHEJ DNA repair of double-strand DNA breaks (DSBs), and also in the protection of telomeres (Walker et al., 2001; Fell and Schild-Poulter, 2015). Although the genetic loss of Ku in mice is associated with genomic instability and increased incidence of lymphomas (Nussenzweig et al., 1996; Gu et al., 1997; Li et al., 1998, 2007), there has been presently no hereditary disease linked to the loss or inactivating mutations of the genes coding for Ku70 (*XRCC6*) or Ku86 (*XRCC5*) in humans, implying an essential function for Ku in human cell viability (Li et al., 2002; Fattah et al., 2008). To date, only infrequent rate of mutations of *XRCC6* or *XRCC5* (0.96 and 1.1% of AACR GENIE cases, respectively), has been reported (AACR Project Genie Consortium, 2017; Printz, 2017). Yet, Ku has been found frequently dysregulated in tumors (Komuro et al., 2002; Mazzarelli et al., 2005; Korabiowska et al., 2006; Abdelbaqi et al., 2013; Wang et al., 2015, 2018; Zhu et al., 2016).

In this study, addressing the cellular dynamics of the Ku70 conditionally-null HCT116 cells, we discovered that loss of Ku proteins is associated with the emergence of parasite-like cell types that are able to invade neighboring cells. Our study provides evidence for a novel paradigm of cell survival in human cancer cells that is characterized with the parasite-like host cell exploitation. Supported by the live-cell imaging studies, our data suggest that the cells challenged with a life-threatening Ku deficiency acquire a parasitic lifestyle by which Ku-deficient cells invade neighboring cells and gain access to the building blocks for maintenance of their survival.

Our study showed that the deletion of *XRCC6* gene (codes for Ku70 protein) in HCT116 Ku70^f/–^ cells caused a massive cell death, as reported earlier (Fattah et al., 2008). However, the tracking of HCT116 Ku70^f/–^ cells following the induction of the conditional loss of Ku70 revealed an emerging cell population that accounts for only 10–20% of the initially seeded cells at day 10 of the 4-OHT treatment. Of note, the difference between our observations of the surviving fraction of the Ku70-conditionally null cells and the previous study reporting the massive cell death in Ku70-null cells (Fattah et al., 2008) may arise from the implementation of a conditional gene deletion system in this study vs. the application of an acute gene deletion methodology in the earlier study. It is likely that the depletion of Ku70 in our conditional system does not occur in a synchronized manner, and does not cause an immediate loss of Ku70 in each cell. As a result, a moderate, but progressive shortage of Ku70 prior to its absolute loss provides sufficient time for these cells to acquire the adaptive defense mechanism of host cell utilization. It is probable that the surviving fraction of Ku70-null HCT116 cells at day 10 (10–20% of initially seeded cells) has already developed the mechanism of survival by adopting some features of the parasitic mode of life.

The data presented here provide a proof of principle for an extreme cellular adaptation to the severe deficiency of a vital protein whose function is indispensable for survival. Further research is required to investigate whether other types of cellular stress such as viral/cellular oncogenes, nutritional starvation, or chronic exposures to chemicals induce parasitic cell invasions in human cells, and furthermore, whether the cellular dynamics of Ku protein are restrained by those stress conditions. Moreover, these findings raise an important question whether Ku malfunction is a possible trait of some cancer cells in which the host cell invasion and/or host cell-dependent cell proliferation is involved in the development of metastatic cancer phenotypes. Following this idea, we identified a medulloblastoma cell line, DAOY cells, that shows similar characteristic of Ku70^null^ HCT116 cells such as giant cells surrounded by smaller cells, and “attacking cells” penetrated into the host cell membranes (Figure 3A). The immunofluorescence analyses of DAOY cells demonstrated that both Ku70 and Ku86 proteins are exclusively localized in the cytoplasm of DAOY cells, while both proteins are excluded from the nuclei of these cells (Figure 3B). Given that Ku70/Ku86 heterodimer is a DNA binding protein that is mainly localized in the nuclei of cells (Walker et al., 2001; Fell and Schild-Poulter, 2015), our findings suggest that Ku protein is mislocalized in DAOY cells, indicating that DAOY cells carry a Ku malfunction in regards to their nuclear functions in DNA repair and telomere protection. Thus, DAOY cells originally derived from a medulloblastoma patient may represent an example of Ku deficiency that is caused by cellular mislocalization, and that may be associated with the parasitic cell phenotype observed in this study.

Considering the formation of multiple vesicular assemblies at the apex of membrane protrusions and their subsequent transportations into the cell body through tubular membrane structures in Ku70^null^ HCT116 cells (Supplementary Movie 5), it would be of great interest to investigate if Ku70^null^ cells are able to take up freely available cellular constituents, for instance extracellular DNA, released from dead cells, and reconstitute and utilize them as cellular building blocks, which highlights a potential recycling system for vital resources such as genetic material.

Another unexpected activity observed in Ku70^null^ HCT116 cells was that the cells exhibiting severe genomic instability such as cells with multiple fragmented nuclei were able to dynamically expand their extensive membrane protrusions to long distances (Supplementary Movie S10). These observations indicate that cells appear to be polyploid with multiple fragmented nuclei can still be metabolically active and may support the survival of the cell population, perhaps by serving as host with a task of supply depository.

Finally, we report here that loss or malfunction of Ku protein leads to a parasitic lifestyle in human cancer cell lines, HCT116 and DAOY cells. Our observations provide evidence for a new mechanism of cellular adaptation as a defense system to severe genomic stress caused by a functional loss of a vital protein, Ku70. Thus, our study brings a new concept for understanding the mechanisms of cancer cell evolution, and offers a radical look at the current view of cancer heterogeneity, metastasis and drug resistance.

## Data Availability Statement

The original contributions presented in the study are included in the article/Supplementary Material, further inquiries can be directed to the corresponding author/s.

## Author Contributions

NS and OS conceptualized the research program and conducted experiments, analyzed the data and wrote the manuscript. OS constructed Palm-EGFP and Palm-tdTomato reporter plasmids and conducted live-cell imaging studies and confocal microscope analyses. Both authors contributed to the article and approved the submitted version.

## Conflict of Interest

The authors declare that the research was conducted in the absence of any commercial or financial relationships that could be construed as a potential conflict of interest.
