# Supplementary material for: Deficiency of Ku Induces Host Cell Exploitation in Human Cancer Cells
Source: Front Cell Dev Biol. 2021 Mar 29;9:651818. doi: 10.3389/fcell.2021.651818 (PMC8040742; doi:10.3389/fcell.2021.651818)
Supplement: Supplementary file 11 [file Data_Sheet_1.pdf]

# Supplementary Materials for

## Deficiency of Ku induces host cell exploitation in human cancer cells

O. Saydam[1] and N. Saydam[2]*

*Correspondence to: nurtensaydam@yahoo.com

**This PDF file includes:**

**Supplementary Movie Captions**
Supplementary Movie 1 to Supplementary Movie 10
**Supplementary Figure Legends**
Supplementary Figure S1A-D (Related to Movie 1)
Supplementary Figure S2A and B (Related to Movie 2)
Supplementary Figure S3A and B (Related to Movie 4)
Supplementary Figure S4A-H (Related to Movie 6)
Supplementary Figure S5A-E (Related to Movie 7)
**Table S1**

**Supplementary Movie Captions**

**Supplementary Movie 1. Ku70 conditionally-null HCT116 cells exploit neighboring cells.** Two small cells exited from a giant cell. One of them (tracked by an arrow) invaded two different neighboring cells in sequence, and turned into a free-living single cell at the end of the movie. Scale bar, 25 µm.

**Supplementary Movie 2. Dynamic movements of a parasitic Ku70$^{null}$ cell within the host.** A Ku70$^{null}$ HCT116 cell dynamically moving within the host was released into the extracellular area upon the host cell death. Scale bar, 25 µm.

**Supplementary Movie 3. Dynamic interaction of a Ku70$^{null}$ mitotic HCT116 cell with the host cell.** A rounded cell (arrow tracked) was located at the membrane of a giant cell (arrow tracked) and underwent into mitosis. Upon failed cytokinesis, three daughter cells were joined together and started to interact with the giant cell body/nuclei as recorded via the dynamic membrane motions of the rounded cell. Scale bar, 25 µm.

**Supplementary Movie 4. Faulty cell division within the host, and cytoplasm adoption of Ku70$^{null}$ HCT116 cells.** An invading Ku70$^{null}$ HCT116 cell revealed two daughter cells/nuclei within the host, but failed to segregate and joined together while exiting the host cell. Upon releasing from the host, this cell adopted cytoplasm from two neighboring cells, and turned into a free-living single cell. Scale bar, 25 µm.

**Supplementary Movie 5. Formation of newly assembled intracellular bodies at the tips of the membrane protrusions in Ku70$^{null}$ HCT116 cells**. A giant Ku70$^{null}$ HCT116 cell shows the formation of some intracellular bodies at the tips of the large membrane protrusions. Those newly formed structures resembling micronuclei were being transported in single file via long and narrow tubes of the plasma membrane in the direction of the cell nuclei. Scale bar, 25 µm.

**Supplementary Movie 6. Parasitic host cell invasion in DAOY cells.** The Palm-EGFP expressing DAOY cells were subjected to live-cell imaging. DAOY cells were recorded to invade and exit from neighboring cells successively, in a period of 48 h. Scale bar, 25 µm.

**Supplementary Movie 7. Dynamic interaction between a parasitic DAOY cell and the host cell.** A mitotic-like DAOY cell, marked with an arrow, was recorded as it was actively rotating on the body of a giant cell. Upon the death of the giant cell, the rotating cell invaded and translocated into a new host cell. Scale bar, 25 µm.

**Supplementary Movie 8. Faulty cytokinesis in DAOY cells.** A cell with two nuclei underwent mitosis. Daughter cells failed to segregate and formed a multinucleated cell. Scale bar, 25 µm.

**Supplementary Movie 9. Host cell exploitation in DAOY cells expressing inducible Ku70-shRNAs.** DAOY cells expressing Palm-EGFP reporter (green) and an inducible shRNA system targeting Ku70 (red) show parasitic cell invasions. Scale bar, 25 µm.

**Supplementary Movie 10. Dynamic movement of a Ku70$^{null}$ HCT116 cell carrying multiple fragmented nuclei.** A Ku70$^{null}$ HCT116 cell showing severe genomic instability was able to actively expand to long distances with extensive membrane protrusions. Scale bar, 25 µm.

**Supplementary Figure Legends**

**Supplementary Figure 1 (Related to Movie 1). Ku70 conditionally-null HCT116 cells exploit neighboring cells.** HCT116 Ku70$^{f/-}$ cells were infected with lentiviruses expressing H2B-EGFP (green) or H2B-mRFP (red). Cells were propagated for one week and Ku70$^{f/-}$ cells infected with second round of lentiviruses expressing either Palm-EGFP or Palm-tdTomato. One week later, Ku70$^{f/-}$ cells were treated with 10 nM 4-OHT for five days. One day before the live-imaging analysis, the Ku70$^{null}$ cells expressing H2B-EGFP+Palm-EGFP were co-cultured (1:1 ratio) with the Ku70$^{null}$ cells expressing H2B-mRFP+Palm-tdTomato.

**(A) Budding-like cell detachment and exit of Ku70-deficient cells from a host cell.** Two cells visualized inside a giant cell are marked with yellow arrows at t=0:00:00 (t represents actual time point recorded in the course of the live-cell imaging for 48 h). At t=1:05:02, these two cells are located at the close proximity to the host cell membrane, where the distortions of the host cell membrane in those sites are visible. The exit of the first cell from the host can be tracked starting at t=1:30:02 through t=2:05:01. The "budding" of the second cell, while causing a visible distortion in the host cell membrane, becomes evident at t=2:05:01 and t=3:25:02, followed by its detachment from the host membrane at t=3:35:02. Scale bar, 25µm.

**(B) Host cell invasion by Ku70-deficient cells.** The cell (expressing H2B-GFP reporter) departed from the giant host cell firstly, as described in (A), was further followed (marked with a yellow arrow). This cell invaded a neighboring cell (H2B-GFP-labelled) at t=16:25:02, and entered into the host cell completely at t=18:05:01, at which the host cell was surrounded by a bright circle that is a typical view of the cell membrane visualized by a microscope. This host cell then underwent into cell division (t=18:15:02), yielding two daughter cells. Subsequently, one of those cells died (red arrow), while the other cell invaded a neighboring cell, which is red labelled with the H2B-mRFP expression, at t=20:05:02 through t=22:20:02. In this specific host cell invasion example, we were able to visualize both the green invading cell nucleus and the red host cell nucleus in one single cell body that was surrounded by an intact, bright host cell membrane (t=23:20:02). The invading cell carrying a green nucleus remained inside the host cell (red nucleus) for at least 20h. At t=42:30:02, the invading cell was visualized to form its own cytoplasm, and it fully detached from the host cell and moved away at t=46:15:02. Scale bar, 25µm.

**(C) Phase contrast images of the live-cell imaging presented in (A).**

**(D) Phase contrast images of the live-cell imaging presented in (B).**

**Supplementary Figure 2 (Related to Movie 2). Dynamic movements of a parasitic Ku70$^{null}$ cell within the host.** HCT116 Ku70$^{null}$ cells expressing H2B-EGFP (green) or H2B-mRFP (red) were prepared as described in Suppl. Fig.1, and subjected to the live cell imaging for 48 h. Scale bar, 25µm.

**(A)** A giant cell with red nuclei was detected to carry a smaller cell (red nucleus) within its body at t=00:00 (yellow arrow). Tracking these cells between t=01:55 and t=04:25 showed that the smaller cell was highly motile within the host cell, coming into a continuous close-contact with the host cell nuclei (t=02:25 and t=04:10). At t=04:25, the smaller cell nucleus and the host cell

nucleus became indistinguishable. However, the smaller cell moved to the left side of the host at t=04:40, and it started to exit the host cell by showing its cytoplasm outside of the host while its nucleus was separating from the host cell nucleus (t=04:45). The complete release of the smaller cell from the giant cell was evident at t=04:50 and t=04:55.

**(B)** Phase contrast images of the live-cell imaging presented in (A).

**Supplementary Figure 3 (Related to Movie 4). Faulty cell division of Ku70$^{null}$ HCT116 cells within the host.** An invading Ku70$^{null}$ HCT116 cell revealed two daughter cells/nuclei within the host cell, but failed to segregate and joined together while exiting the host cell. Scale bar, 25µm.

**(A)** A cell expressing H2B-mRFP (yellow arrow) invaded a neighboring cell expressing H2B-EGFP starting from t=01:20 through t=01:20. The invading cell started to reveal two red-labelled nuclei, while it was leaving the host cell (t=04:35 through t=06:20). However, these two nuclei were not segregated into two individual cells. Instead, one of these daughter nuclei was transferred into and merged with the other daughter nuclei during the exit of the invader cell from the host (t=08:40 through t=13:30).

**(B)** Phase contrast images of Movie 4 as presented in (A).

**Supplementary Figure 4 (Related to Movie 6). Parasitic host cell invasion in DAOY cells.** DAOY cells were infected with the Palm-EGFP expressing lentiviruses, and selected with puromycin (1 µg/ml) for 5 day prior to the live-cell imaging. DAOY cells were recorded to invade and exit from neighboring cells successively, in a period of 48 h. Due to the multiple host cell invasion and exit cycles, the images taken from each invasion/exit cycle are presented as separate figures from (A) to (D). Scale bar, 25µm.

**(A) Phase contrast images of DAOY cells subjected to live-cell imaging_Part 1:** Following the cell division, a smaller cell marked with an arrow started to invade the adjacent cell at t=01:04. At t=01:48, both cells were visualized merged together. The cells started to separate between t=02:00 and t=03:04.

**(B) Phase contrast images of DAOY cells subjected to live-cell imaging_Part 2:** Following the separation of these cells, a relatively smaller cell (marked with yellow arrow) started to invade another neighboring cell at t=03:48. The invading cell was observed to be merged with the cytoplasm of the host cell, while both cell nuclei were in close proximity, at t=04:52. The invading cell then moved through the cytoplasmic arm of the host cell (t=06:24 and t=07:00) and translocated to the adjacent cell at t=07:44.

**(C) Phase contrast images of DAOY cells subjected to live-cell imaging_Part 3:** The invading cell that was translocated to another host cell is marked with an arrow. We noticed that this invading cell likely underwent a cell division at t=10:08 (two smaller daughter cells were marked by an arrow). However, those cells failed to separate and merged back to a single cell, as shown at t=10:44. This cell started to translocate to another adjacent cell at t=12:52.

**(D) Phase contrast images of DAOY cells subjected to live-cell imaging_Part 4:** The translocating (invader) cell was visualized in between the neighboring cells at t=14:16. However, it returned to the previous host cell at t=15:24 and remained in this cell through t=27:56. Note

that the active movements of the invading cell in the host causes severe dislocations of the host cell nuclei throughout Movie 6.

**(E) Merged (phase contrast and fluorescence) images of DAOY cells subjected to live-cell imaging_Part 1.**

**(F) Merged (phase contrast and fluorescence) images of DAOY cells subjected to live-cell imaging_Part 2.**

**(G) Merged (phase contrast and fluorescence) images of DAOY cells subjected to live-cell imaging_Part 3.**

**(H) Merged (phase contrast and fluorescence) images of DAOY cells subjected to live-cell imaging_Part 4.**

**Supplementary Figure 5 (Related to Movie 7). Dynamic interaction between a parasitic DAOY cell and the host cell.** A mitotic-like DAOY cell (yellow arrow) was recorded as it was actively rotating on the body of a giant cell. Upon the death of the giant cell, the rotating cell invaded and translocated into a new host cell. Scale bar, 25μm.

**(A) Phase contrast images of Movie 7.** An actively moving, rounded, DAOY cell was visualized on the body of a large cell (t=00:00). This cell remained on the body of the giant cell for around 26 h (t=26:32), while it was actively rotating and coming into frequent close-contacts with the host cell nucleus. This rounded cell revealed an elongated form at t=23:32, which was still docking into the host cell and circling around its nucleus. This cell was observed to take six turns around the host cell nuclei prior to the translocation to another host cell at t=26:32.

**(B) Fluorescence images of Movie 7 as presented in (A).**

**(C) Merged (phase contrast and fluorescence) images of Movie 7 as presented in (A).**

**(D)** An actively moving, rounded, DAOY cell was visualized on the body of a large cell (t=00:28). This cell revealed to form two separating bodies, similar to mitotic cell division, at t=00:40 through t=00:56. However, starting at t=01:16, these two smaller cell bodies merged into one single cell (t=01:52). Phase contrast images are shown.

**(E)** Similar to (A), the actively moving, rounded DAOY cell was observed to reveal a mitotic-like cell division, forming two cellular bodies at t=03:12. However, these two bodies were not separated in the following time periods, but rather merged into one single cell starting at t=03:16 through t=03:28. Phase contrast images are shown.

**Supplementary Figure S1 (Related to Movie 1). Ku70 conditionally-null HCT116 cells exploit neighboring cells.**

**(A) Budding-like cell detachment and exit of Ku70-deficient cells from a host cell.**

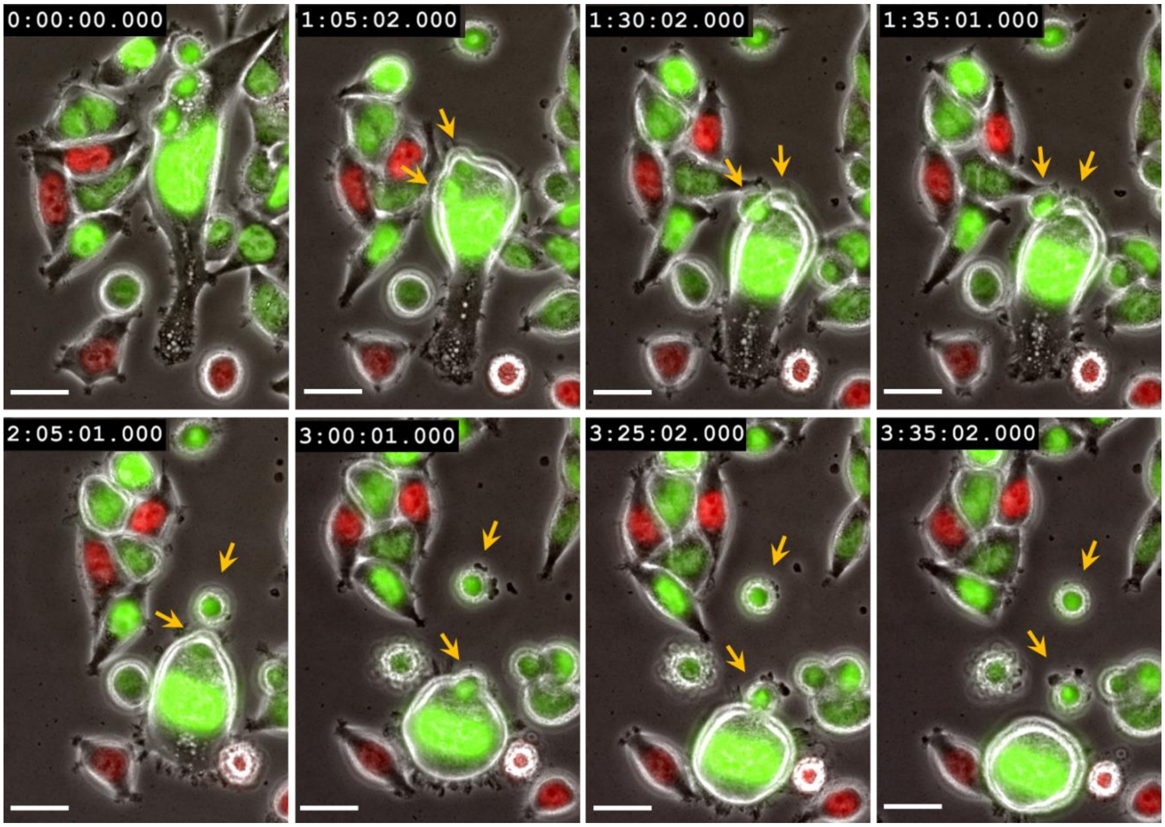

**(B) Host cell invasion by Ku70-deficient cells.**

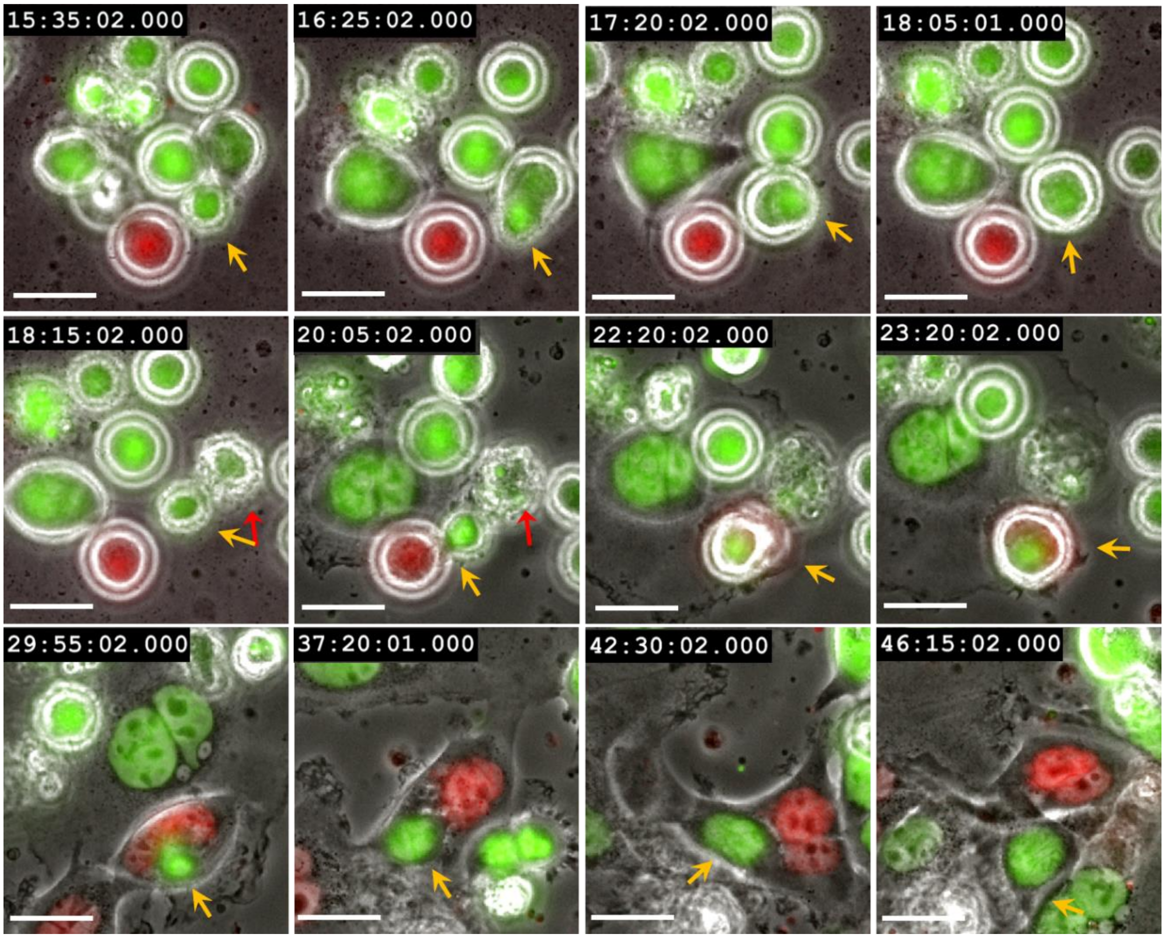

**(C) Phase contrast images of the live-cell imaging presented in (A).**

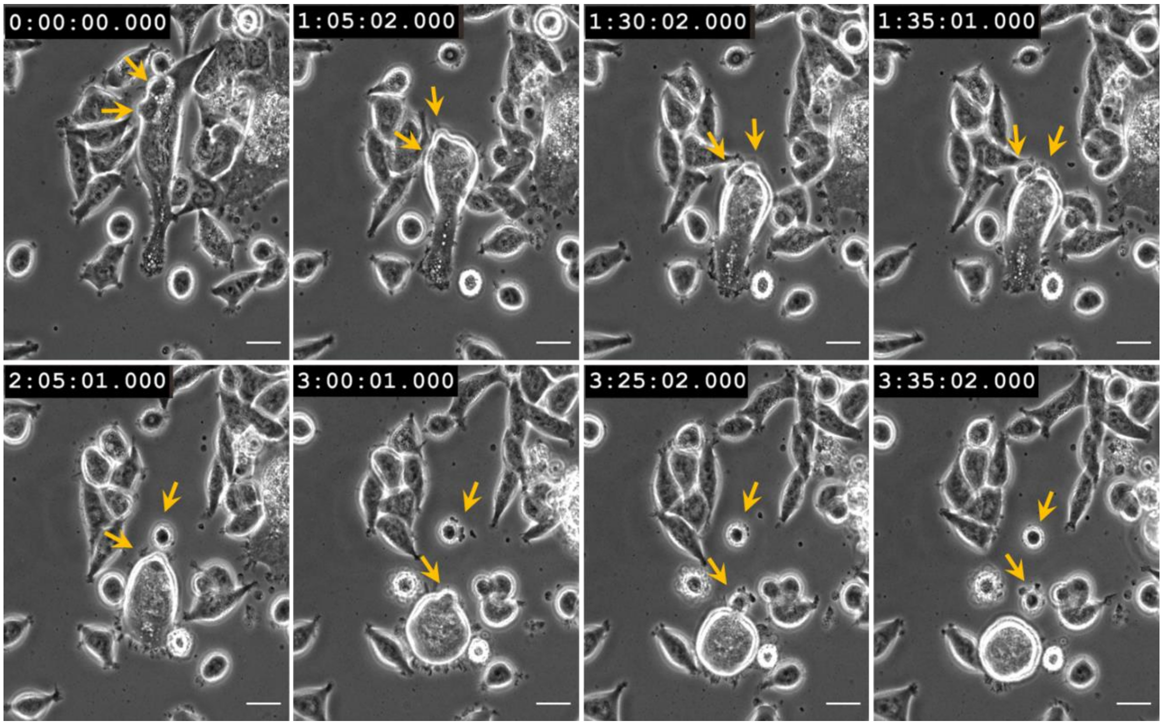

**(D) Phase contrast images of the live-cell imaging presented in (B).**

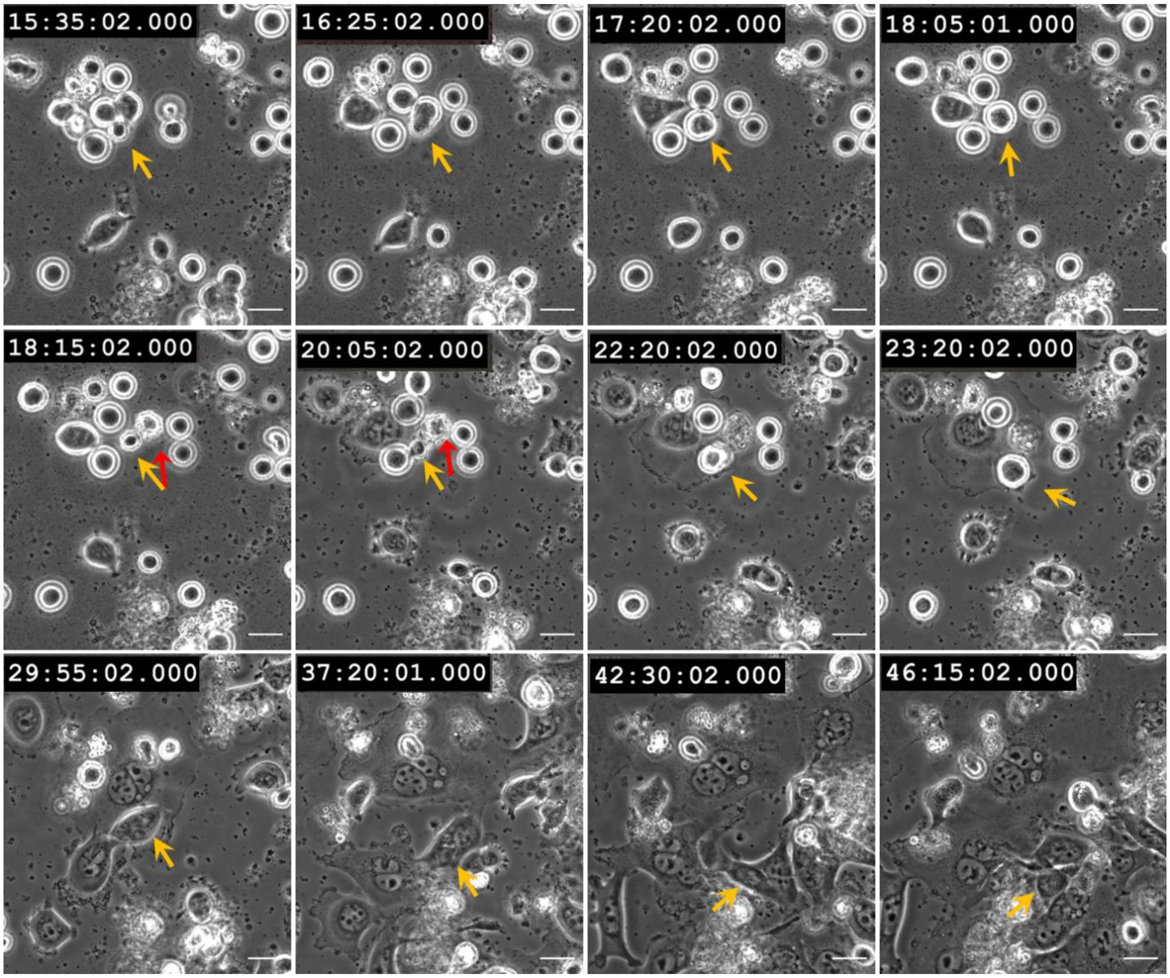

**Supplementary Figure 2A (Related to Movie 2). Dynamic movements of a parasitic Ku70$^{null}$ cell within the host.**

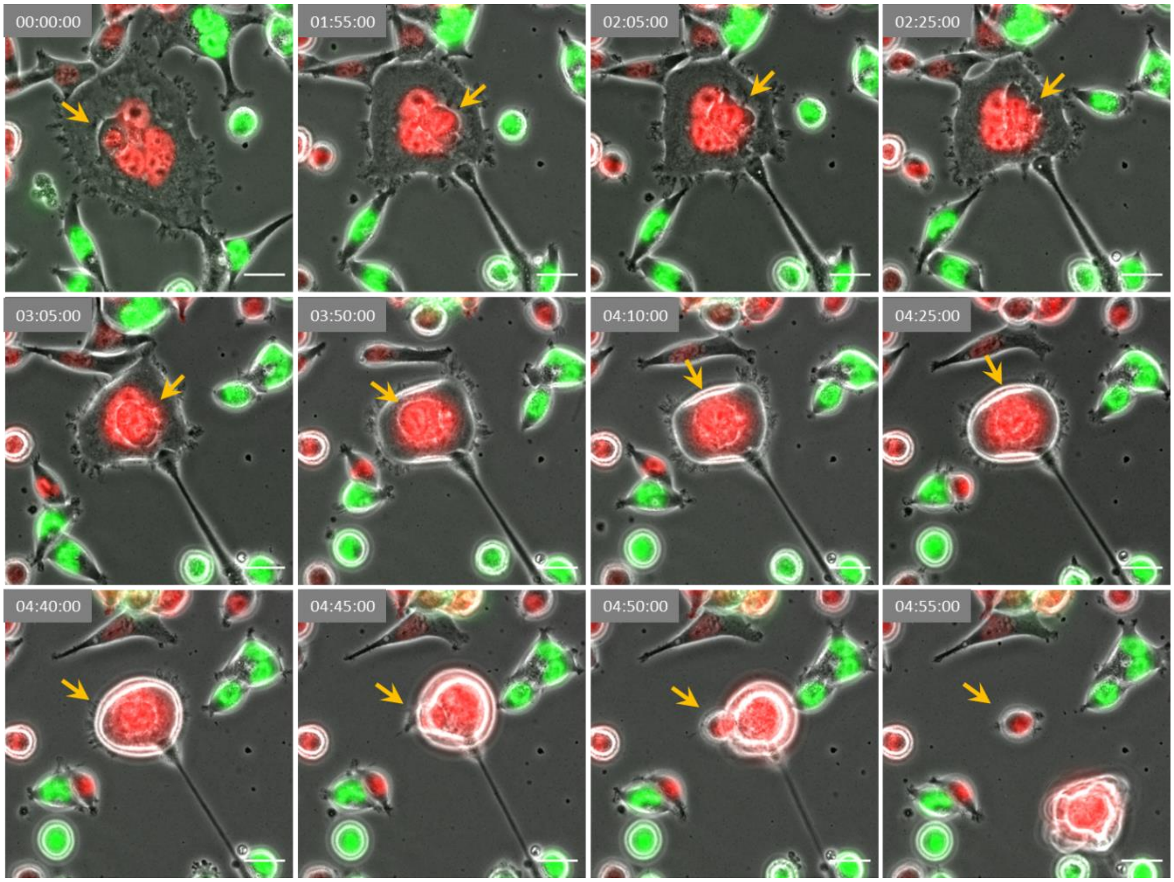

**(B) Phase contrast images of the live-cell imaging presented in (A).**

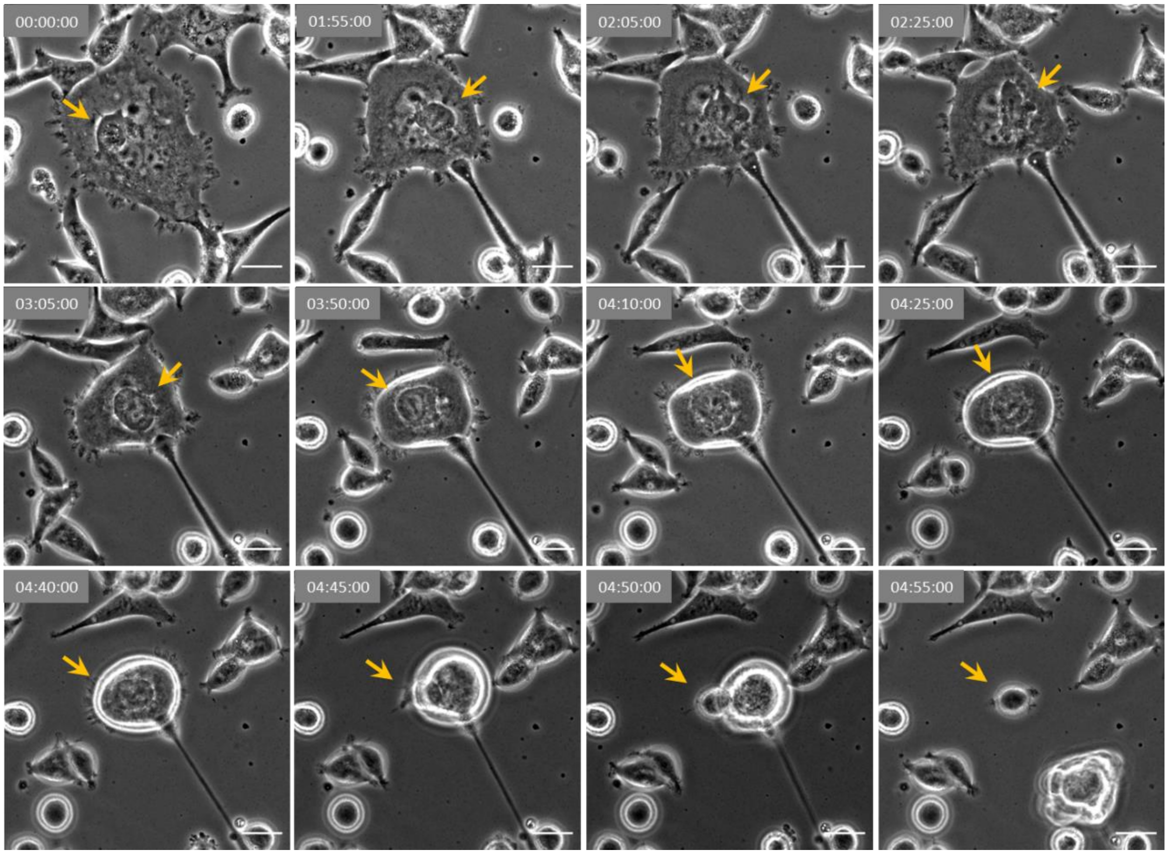

**Supplementary Figure 3A (Related to Movie 4). Faulty cell division of Ku70null HCT116 cells within the host.**

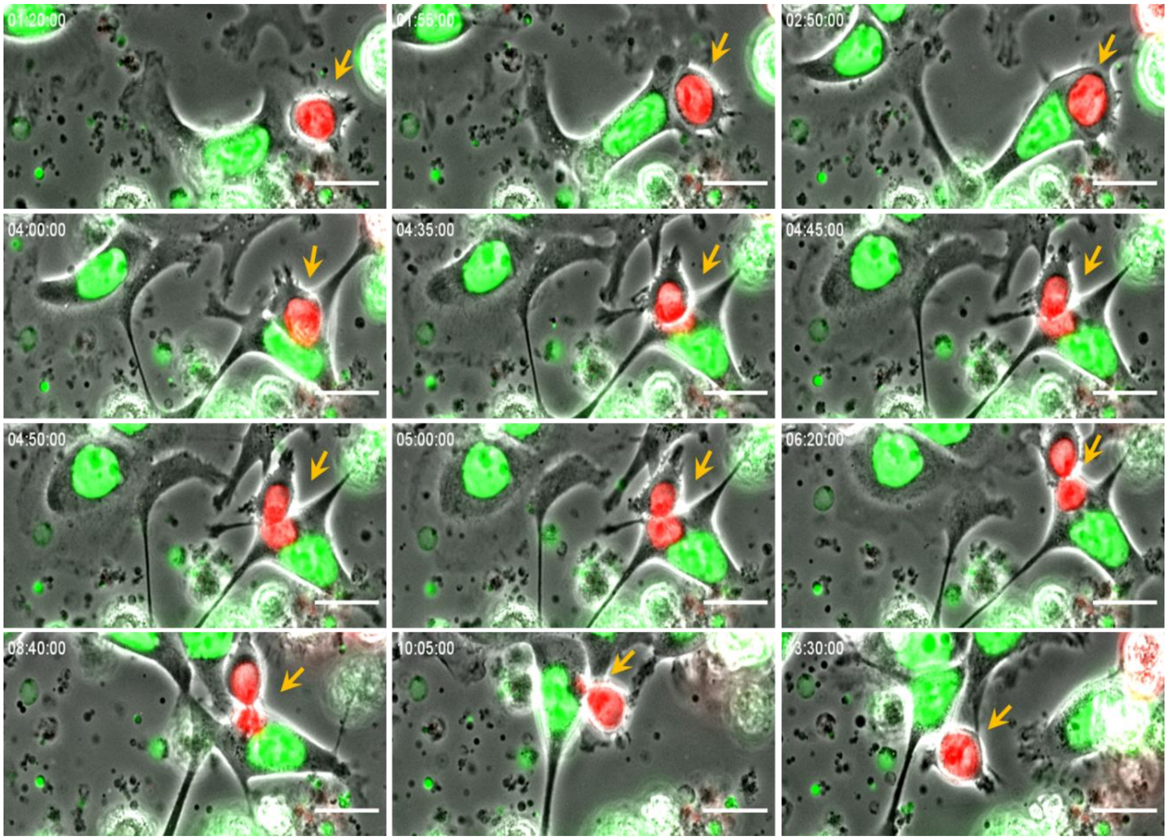

**(B) Phase contrast images of Movie 4 as presented in (A).**

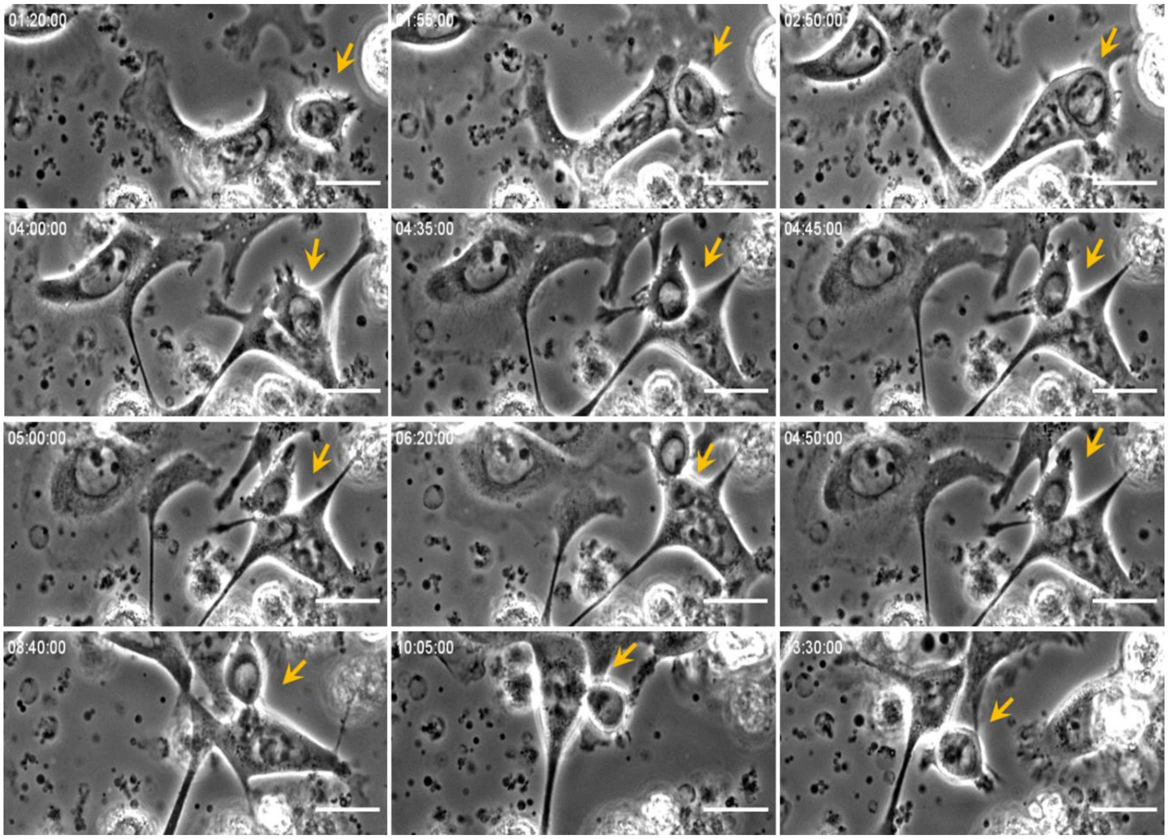

**Supplementary Figure 4 (Related to Movie 6). Parasitic host cell invasion in DAOY cells.**

**(A) Phase contrast images of DAOY cells subjected to live-cell imaging_Part 1**

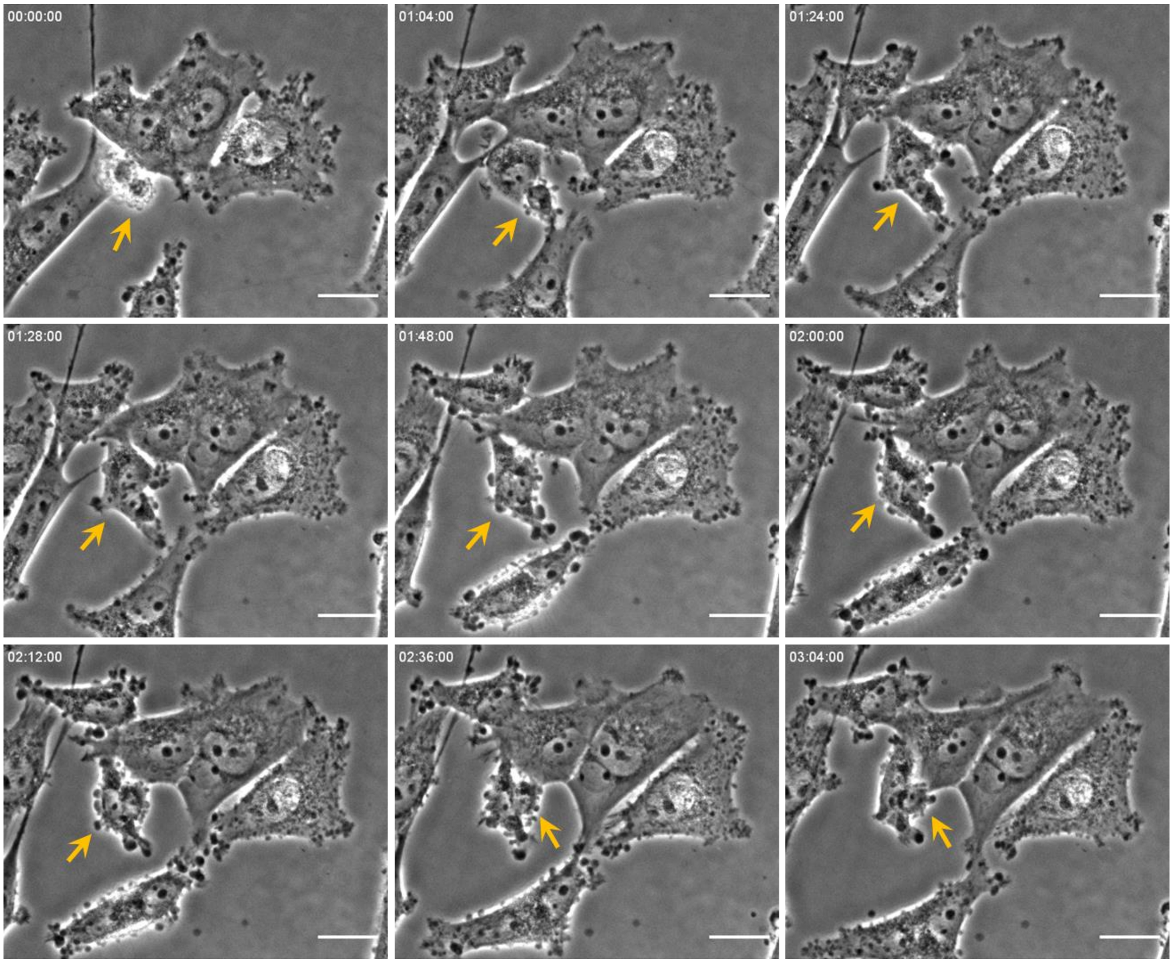

**(B) Phase contrast images of DAOY cells subjected to live-cell imaging_Part 2**

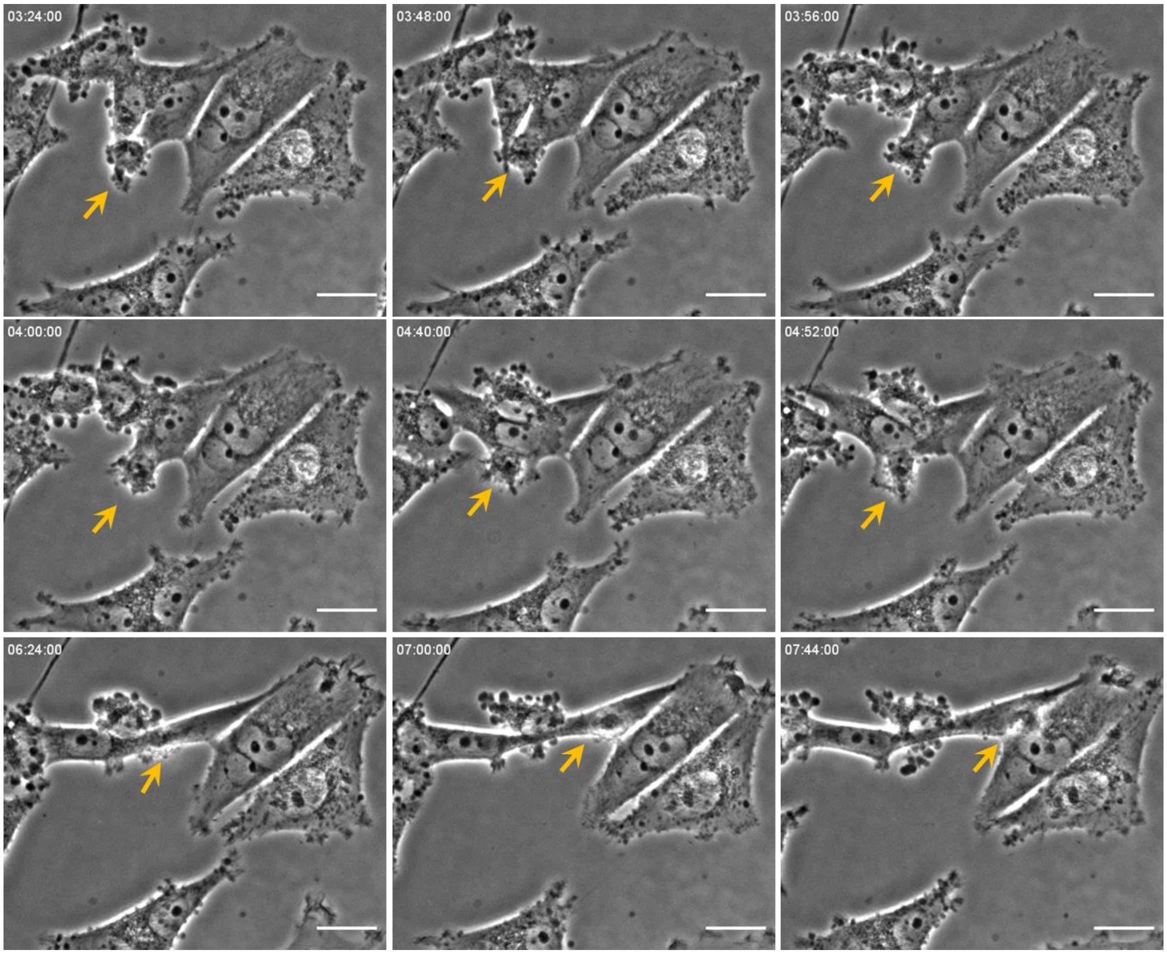

## (C) Phase contrast images of DAOY cells subjected to live-cell imaging_Part 3

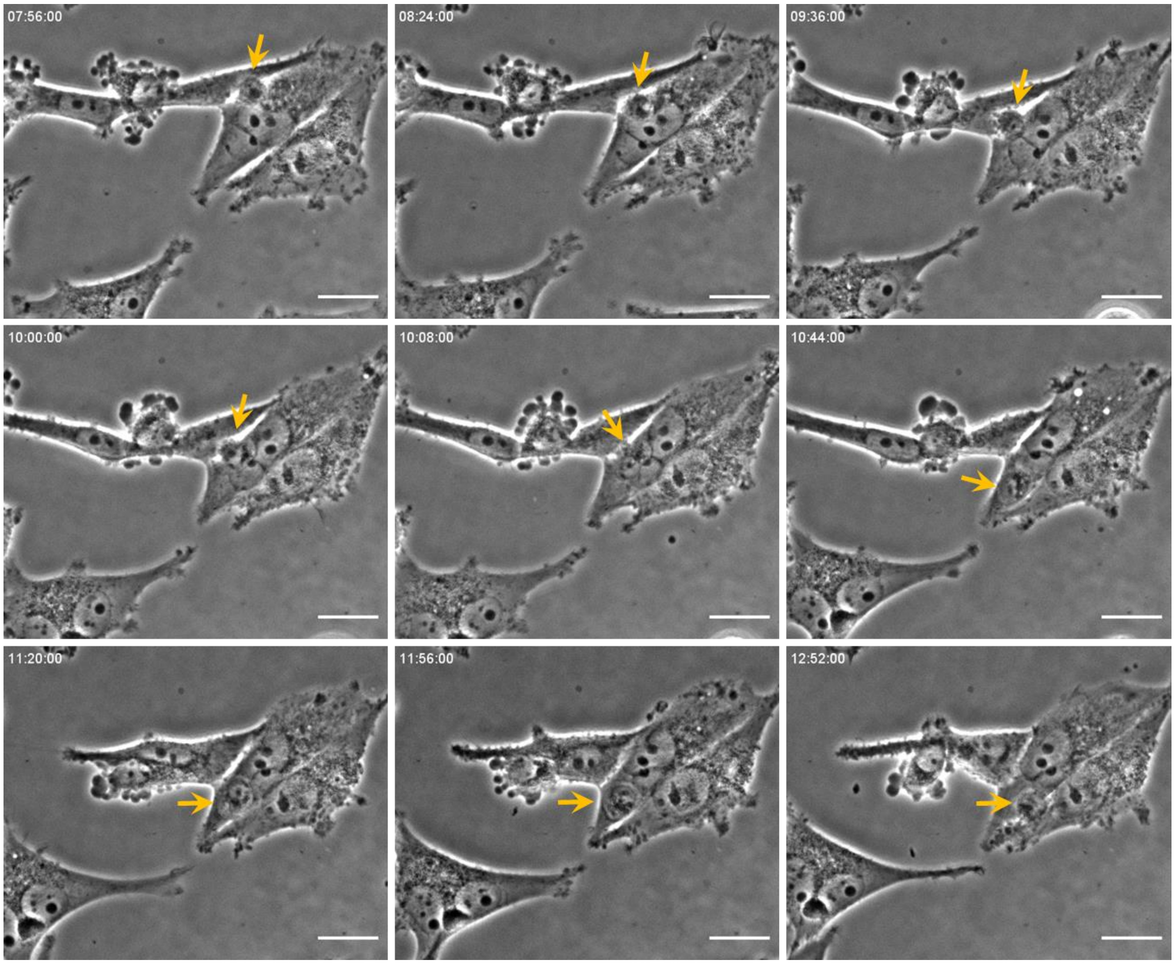

## (D) Phase contrast images of DAOY cells subjected to live-cell imaging_Part 4

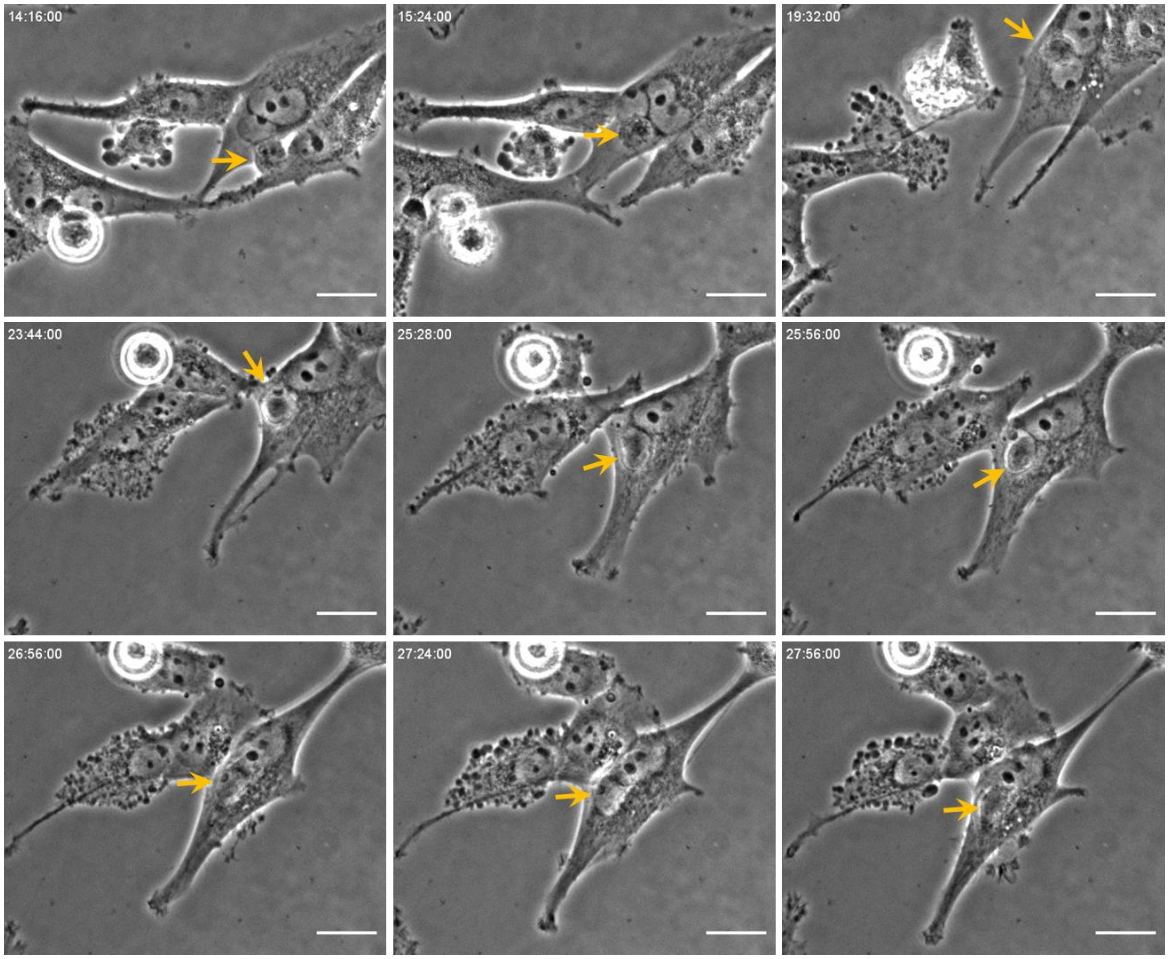

**(E) Merged (phase contrast and fluorescence) images of DAOY cells subjected to live-cell imaging_Part 1**

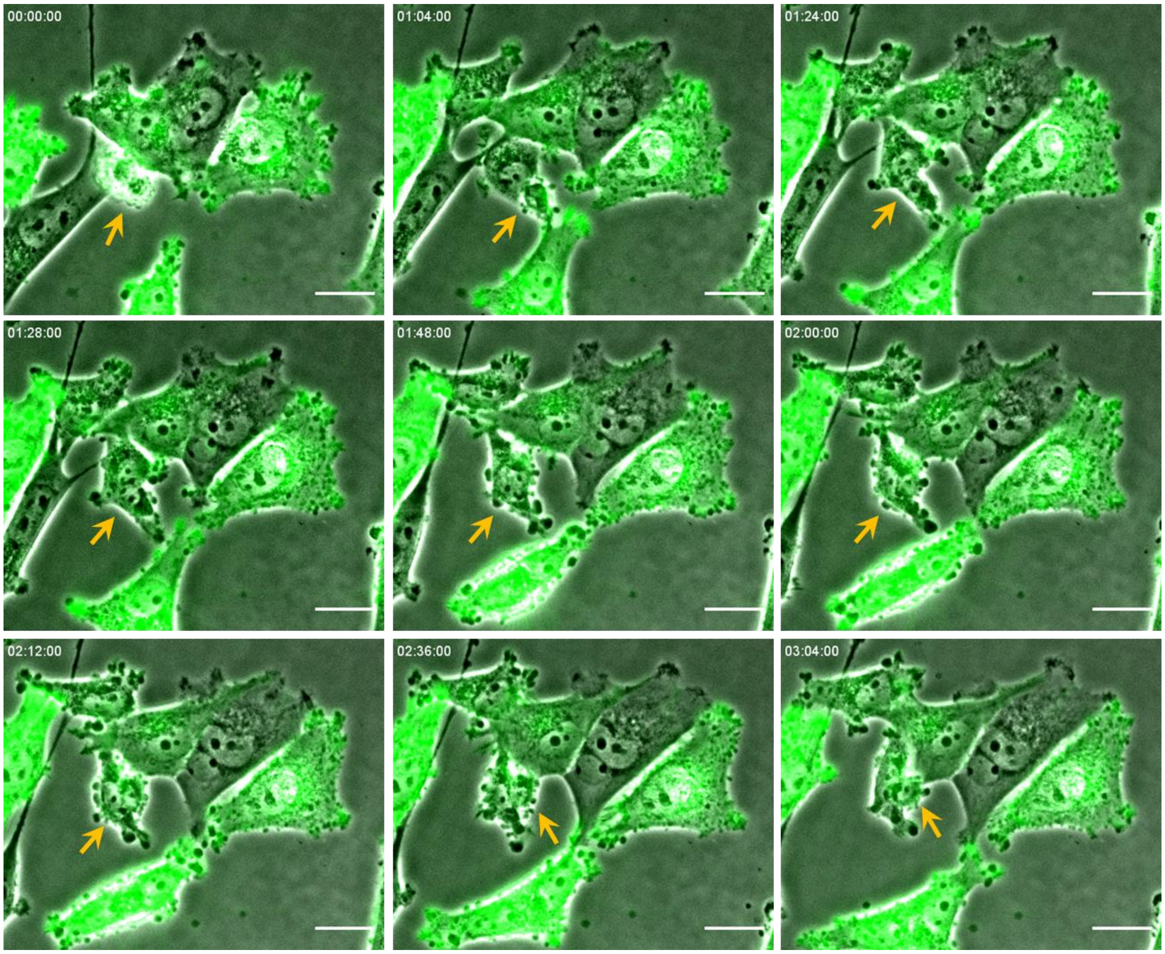

**(F) Merged (phase contrast and fluorescence) images of DAOY cells subjected to live-cell imaging_Part 2**

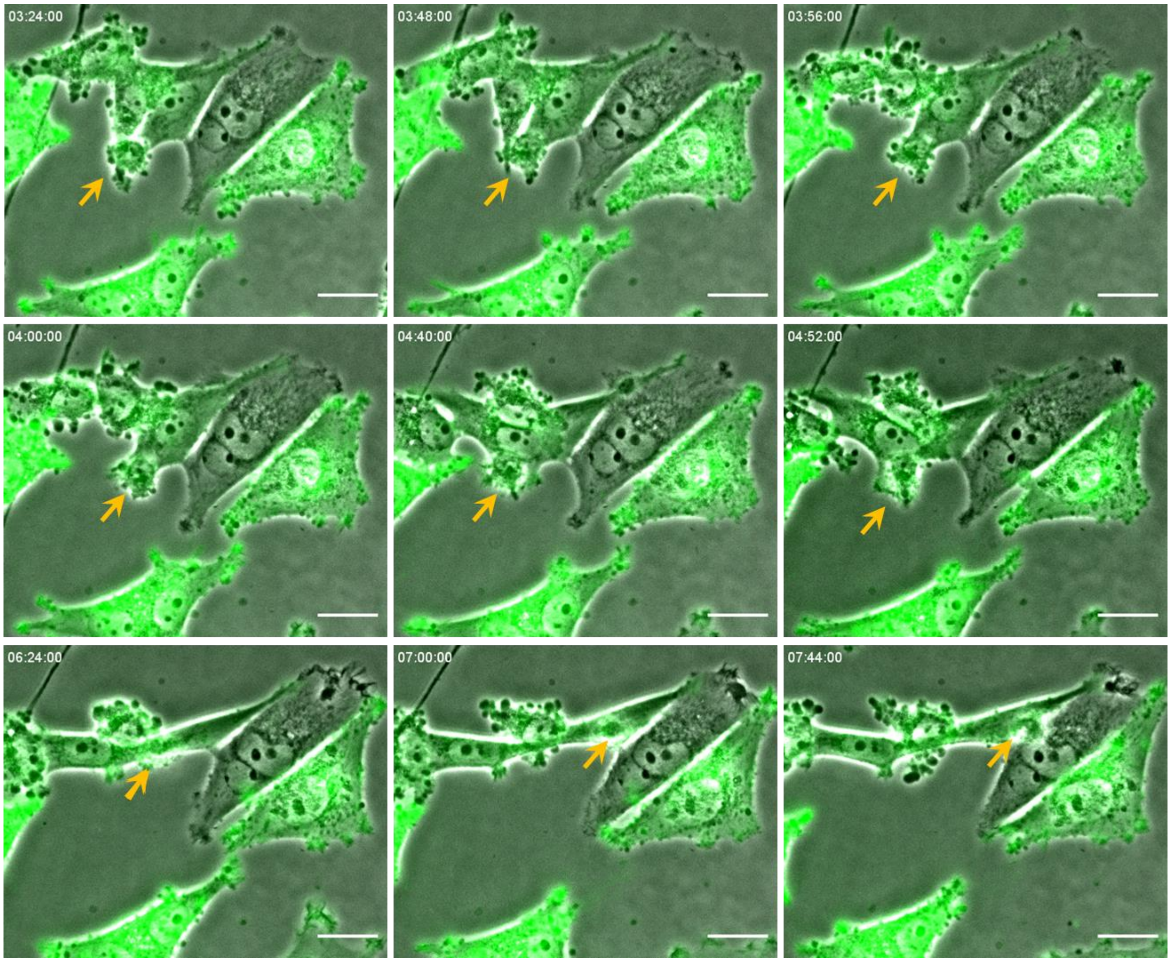

**(G) Merged (phase contrast and fluorescence) images of DAOY cells subjected to live-cell imaging_Part 3**

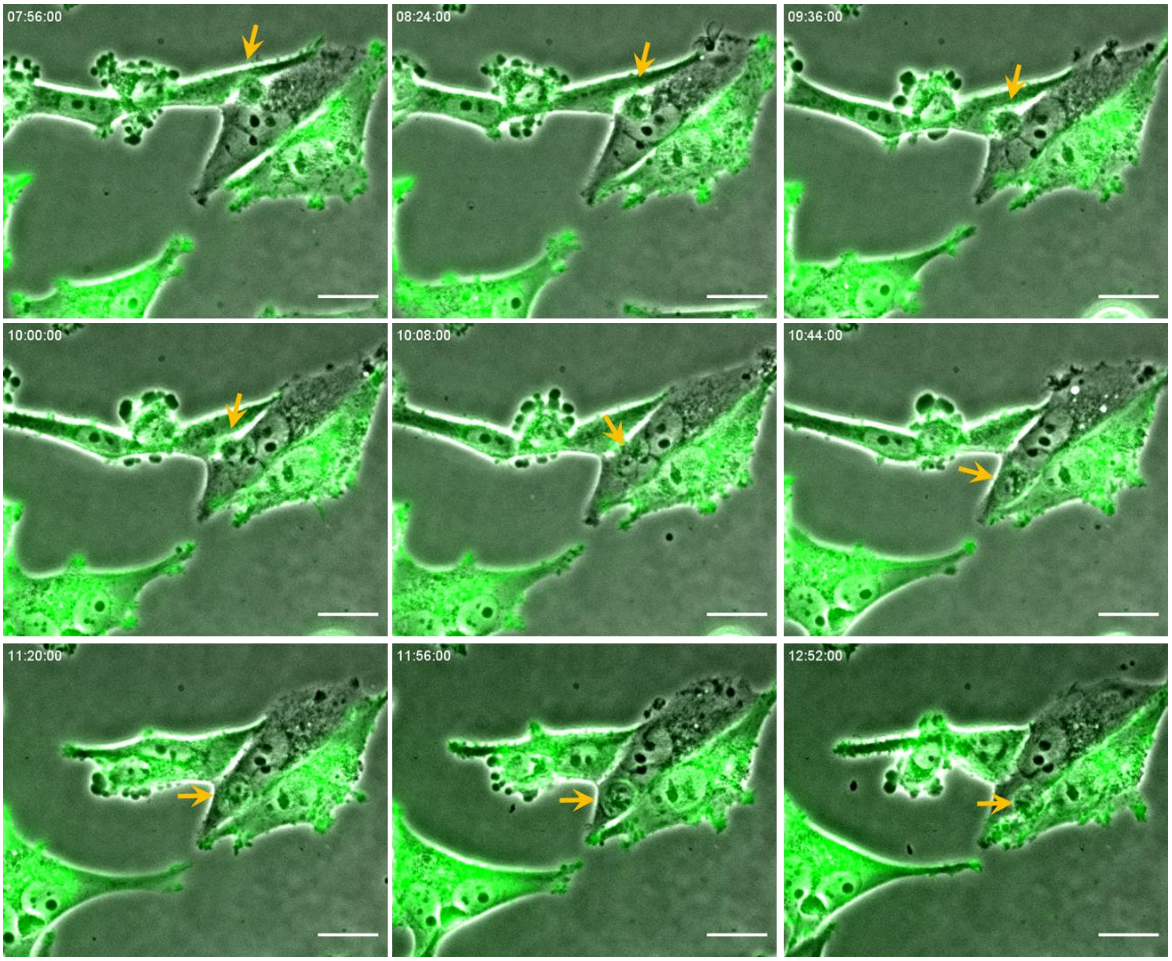

**(H) Merged (phase contrast and fluorescence) images of DAOY cells subjected to live-cell imaging_Part 4**

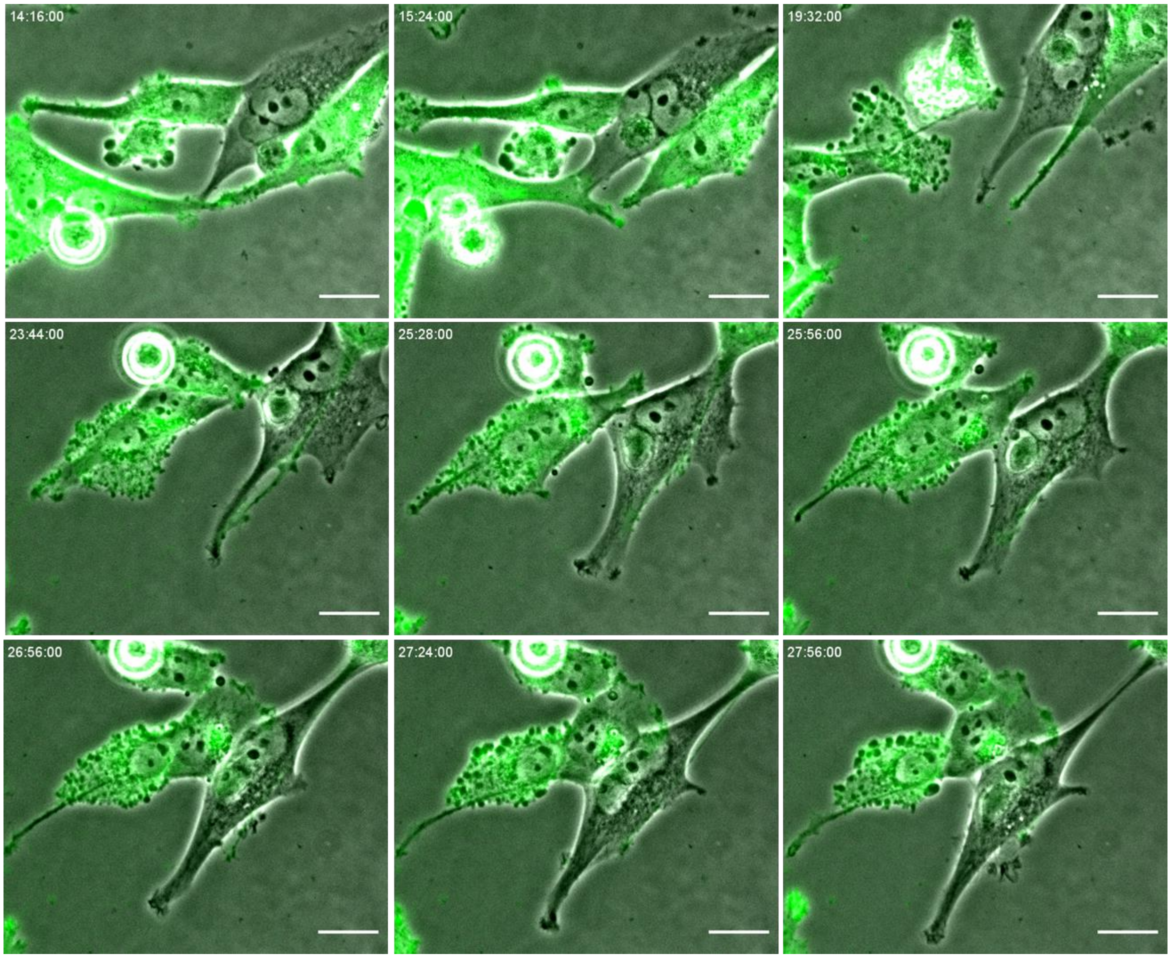

**Supplementary Figure 5 (Related to Movie 7). Dynamic interaction between a parasitic DAOY cell and the host cell.**

**(A) Phase contrast images of Movie 7**

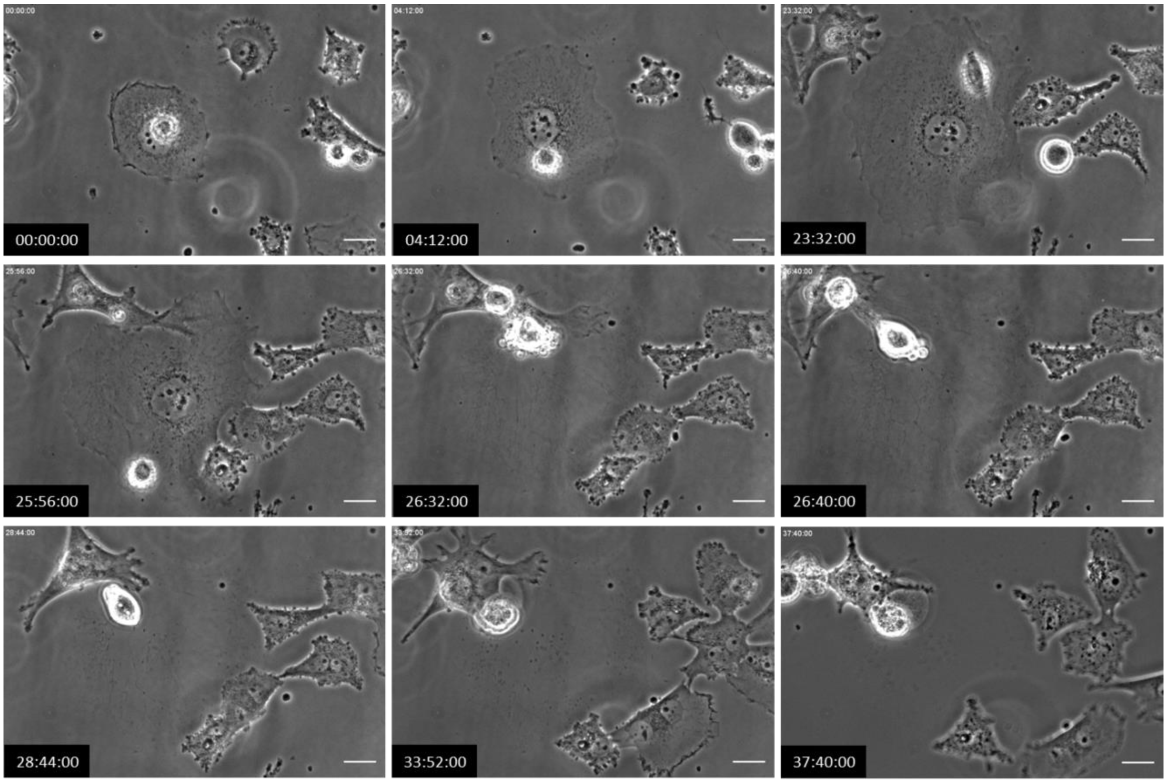

**(B) Fluorescence images of Movie 7 as presented in (A)**

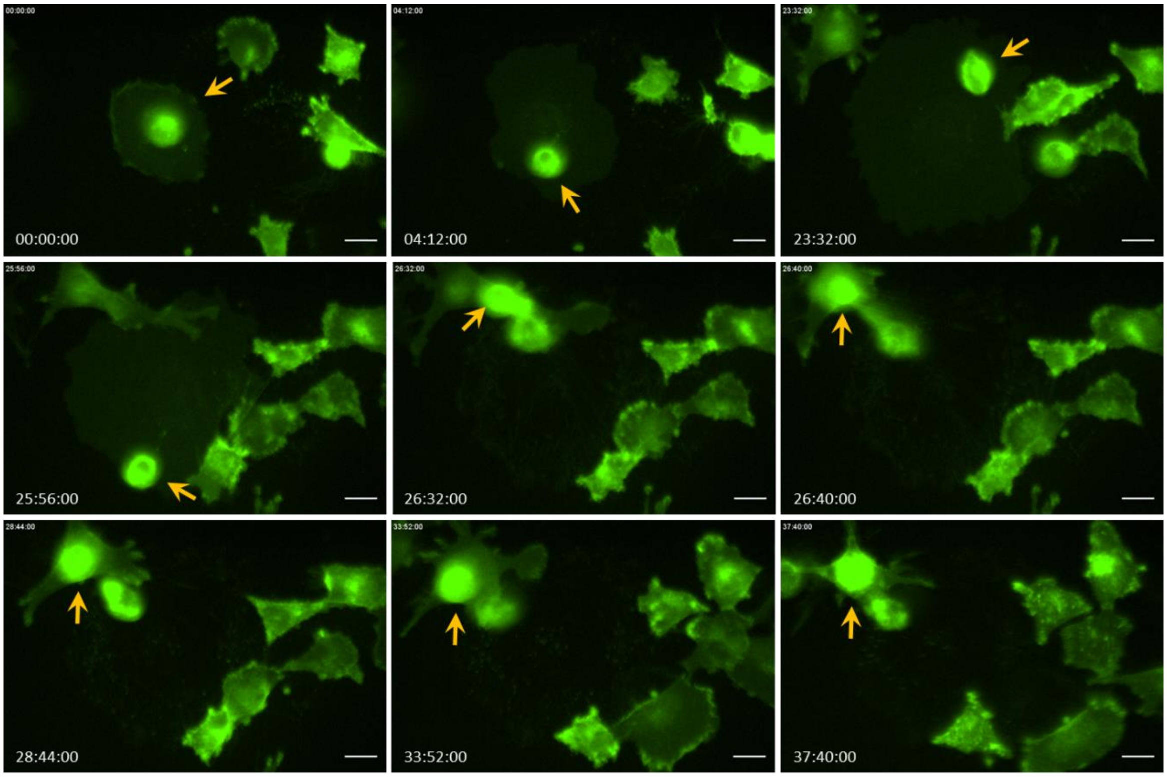

**(C) Merged (phase contrast and fluorescence) images of Movie 7 as presented in (A)**

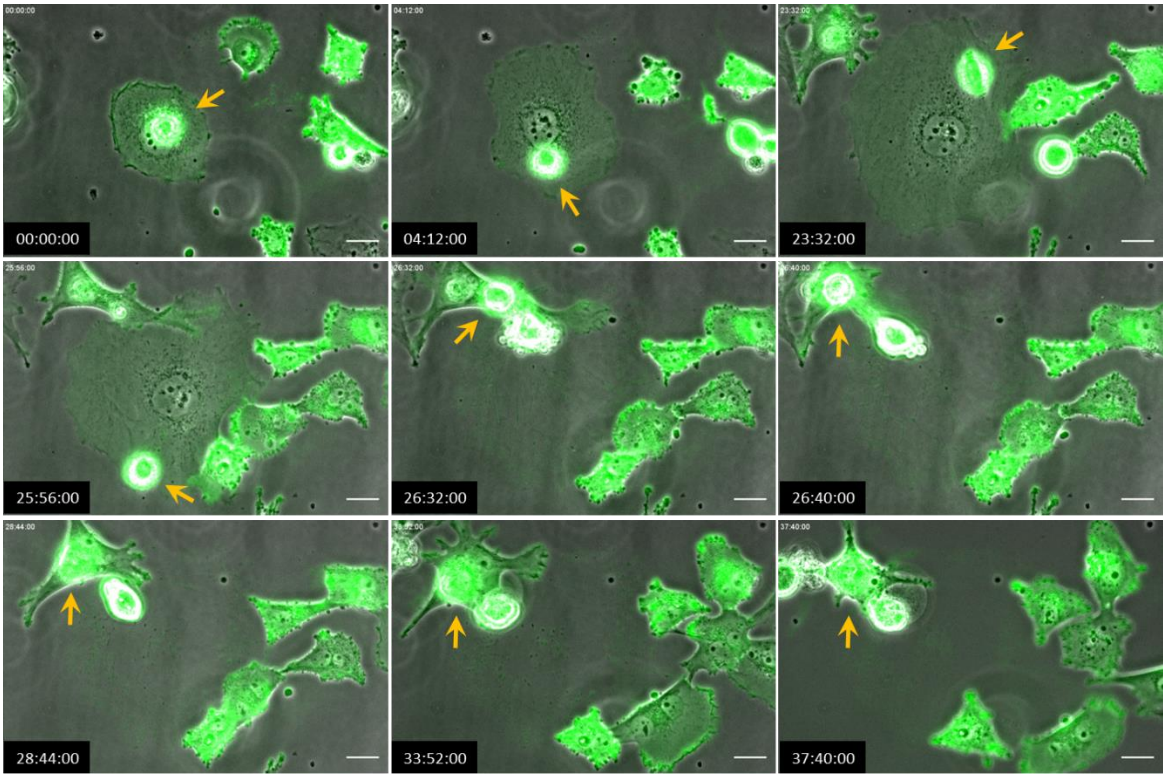

**(D) Formation of daughter cells and segregation defect in DAOY cells (Example 1)**

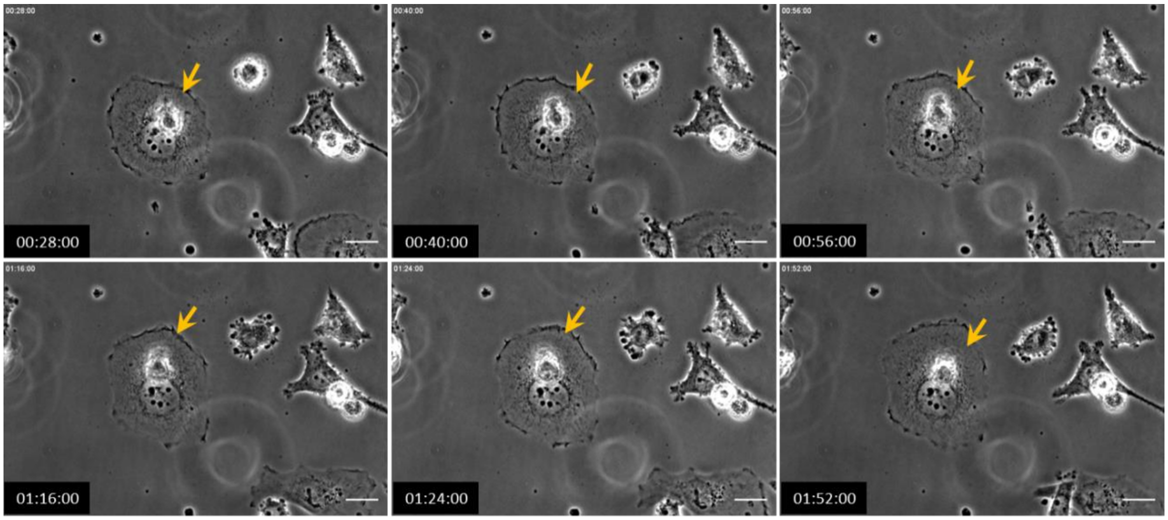


**(E) Formation of daughter cells and segregation defect in DAOY cells (Example 2)**

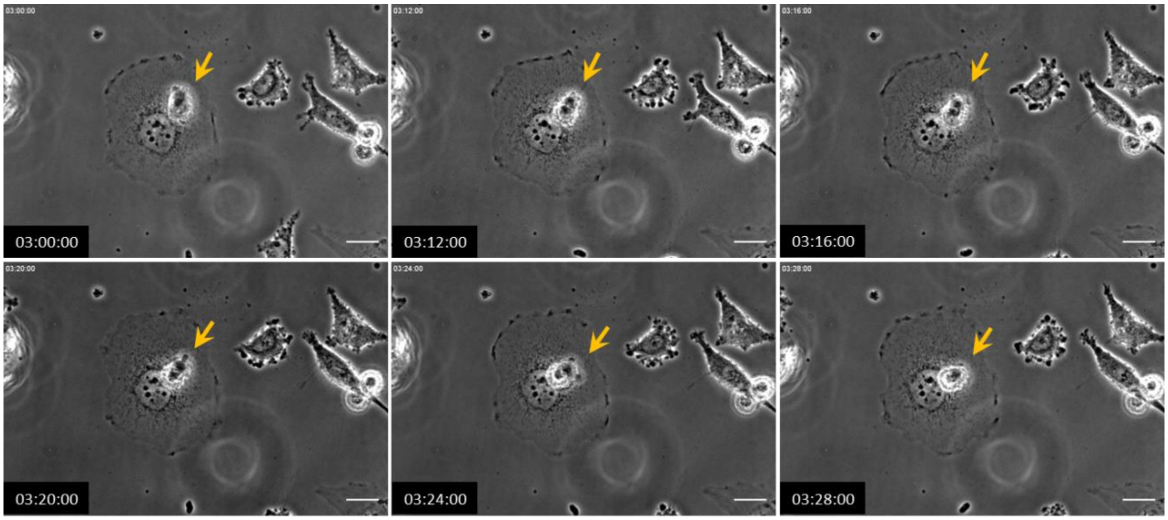

**Supplementary Table 1. List of antibodies used in the study**

| Primary Antibodies | Supplier | Species | Type | Dilution | Reference |
|---|---|---|---|---|---|
| **Ku70 (H-308)** | Santa Cruz | Rabbit | polyclonal | 1:1000 WB<br>1:100 IF | sc-9033 |
| **Ku70** | Santa Cruz | Mouse | monoclonal | 1:1000 WB<br>1:100 IF | sc-17789 |
| **Ku86 (B-1)** | Santa Cruz | Mouse | monoclonal | 1:1000 WB<br>1:100 IF | sc-5280 |
| **β-Actin (AC-15)** | Novus | Mouse | monoclonal | 1:10,000 WB | NB600-501 |
| **Phospho-ERM** | Cell Signaling | Rabbit | monoclonal | 1:1000 WB<br>1:100 IF | #3726 |
| **Secondary Antibodies** | **Supplier** | **Species** | **Type** | **Dilution** | **Reference** |
| **Goat anti-mouse IgG-HRP** | Bio-Rad | Mouse | polyclonal | 1:2000 WB | #170-6516 |
| **Goat anti-rabbit IgG-HRP** | Bio-Rad | Rabbit | polyclonal | 1:2000 WB | #170-6515 |
| **Goat anti-Mouse IgG (H+L) Alexa Fluor 488** | Thermo Fisher Scientific | Mouse | polyclonal | 1:500 IF | #A11029 |
| **Donkey anti-Rabbit IgG (H+L) Alexa Fluor 568** | Thermo Fisher Scientific | Rabbit | polyclonal | 1:500 IF | #A10042 |
